# Global remodeling of the mouse DNA methylome during aging and in response to calorie restriction

**DOI:** 10.1111/acel.12738

**Published:** 2018-03-25

**Authors:** András Sziráki, Alexander Tyshkovskiy, Vadim N. Gladyshev

**Affiliations:** ^1^ Division of Genetics Department of Medicine Brigham and Women's Hospital and Harvard Medical School Boston MA USA; ^2^ Center for Data‐Intensive Biomedicine and Biotechnology Skolkovo Institute of Science and Technology Moscow Russia

**Keywords:** aging, calorie restriction, DNA methylation, entropy

## Abstract

Aging is characterized by numerous molecular changes, such as accumulation of molecular damage and altered gene expression, many of which are linked to DNA methylation. Here, we characterize the blood DNA methylome across 16 age groups of mice and report numerous global, region‐ and site‐specific features, as well as the associated dynamics of methylation changes. Transition of the methylome throughout lifespan was not uniform, with many sites showing accelerated changes in late life. The associated genes and promoters were enriched for aging‐related pathways, pointing to a fundamental link between DNA methylation and control of the aging process. Calorie restriction both shifted the overall methylation pattern and was accompanied by its gradual age‐related remodeling, the latter contributing to the lifespan‐extending effect. With age, both highly and poorly methylated sites trended toward intermediate levels, and aging was accompanied by an accelerated increase in entropy, consistent with damage accumulation. However, the entropy effects differed for the sites that increased, decreased and did not change methylation with age. Many sites trailed behind, whereas some followed or even exceeded the entropy trajectory and altered the developmental DNA methylation pattern. The patterns we observed in certain genomic regions were conserved between humans and mice, suggesting common principles of functional DNA methylome remodeling and its critical role in aging. The highly resolved DNA methylome remodeling provides an excellent model for understanding systemic changes that characterize the aging process.

## 1 INTRODUCTION

Aging is characterized by the gradual reduction in the ability to cope with physiological challenges, which ultimately leads to death (Johnson, Sinclair, & Guarente, 1999). It is clear that the genome defines species lifespan, but the DNA sequence in itself cannot fully explain the aging process. Analyses of epigenetic phenomena, which affect gene expression and chromatin structure and link environmental and genetic factors, may offer a better understanding of the aging process. DNA methylation, histone posttranslational modifications, and microRNA expression show complex changes during aging (Huidobro, Fernandez, & Fraga, 2013). For example, DNA methylation is gradually decreased during aging in different species, including humans, but certain genomic regions, such as CpG islands, are known to gain methylation with age. There is an emerging need for deep analyses of the DNA methylome across ages to define the specific patterns and determine whether the changes are uniform across the genome and its regions, and whether they are consistent during aging. Increased mortality with age in humans supports the idea of acceleration, and this is also consistent with the current understanding of the aging process (Gladyshev, 2016), although experimental analyses are needed to clarify these issues.

Several earlier studies investigated DNA methylation changes during aging in humans (Horvath et al., 2012; Johansson, Enroth, & Gyllensten, 2013; Jones, Goodman, & Kobor, 2015; McClay et al., 2014; Rakyan et al., 2010; Sun et al., 2010). Changes in DNA methylation with age are less clearly understood in mice; yet, mouse studies offer an opportunity to develop a deeper understanding of methylome remodeling due to the availability of genetic and dietary models and amenability for whole‐organism experiments. Previous mouse studies revealed age‐related changes in promoters and increasing methylation of development‐ and differentiation‐related pathways (Maegawa et al., 2010). A global age‐related increase in entropy was also shown (Wang et al., 2017), and dietary restriction was found to influence hepatic genes involved in lipid metabolism‐related pathways (Hahn et al., 2017). Another advantage of mouse studies is that they allow investigating evolutionarily conserved and species‐specific changes during aging and examining the effects of interventions that extend lifespan. Such studies may broaden our understanding of epigenetic regulation of aging and the molecular basis of lifespan‐extending interventions. In this study, we examined these questions in detail by characterizing highly resolved aging‐related changes in the mouse blood DNA methylome.

## 2 RESULTS

### 2.1 Global trends in DNA methylation during aging

We assessed age‐related changes in DNA methylation based on reduced representation bisulfite sequencing (RRBS) of blood of 141 C57BL/6 male mice representing 16 age groups (Petkovich et al., 2017; Table S3). The youngest mice were 3 months old (young adults), and the oldest 35 months old (corresponding to the survival of the remaining 10% animals). After filtering and preprocessing (see Experimental Procedures), the dataset included approximately 800,000 CpG sites present in every sample. We performed multidimensional scaling to investigate global changes in the DNA methylome with age (Figure S1a). The average methylation status of CpG sites was slightly below 0.5 (one sample Student's *t* test, *p*‐value = 1.99 × 10^−134^; Figure S1b), that is, the genome was slightly hypomethylated. Linear regression was then performed, accounting for every site and possible confounding factors. There was a slight, but significant decrease in the global methylome with age (*p*‐value = 6.05 × 10^−152^; Figure S1b).

**Figure 1:**
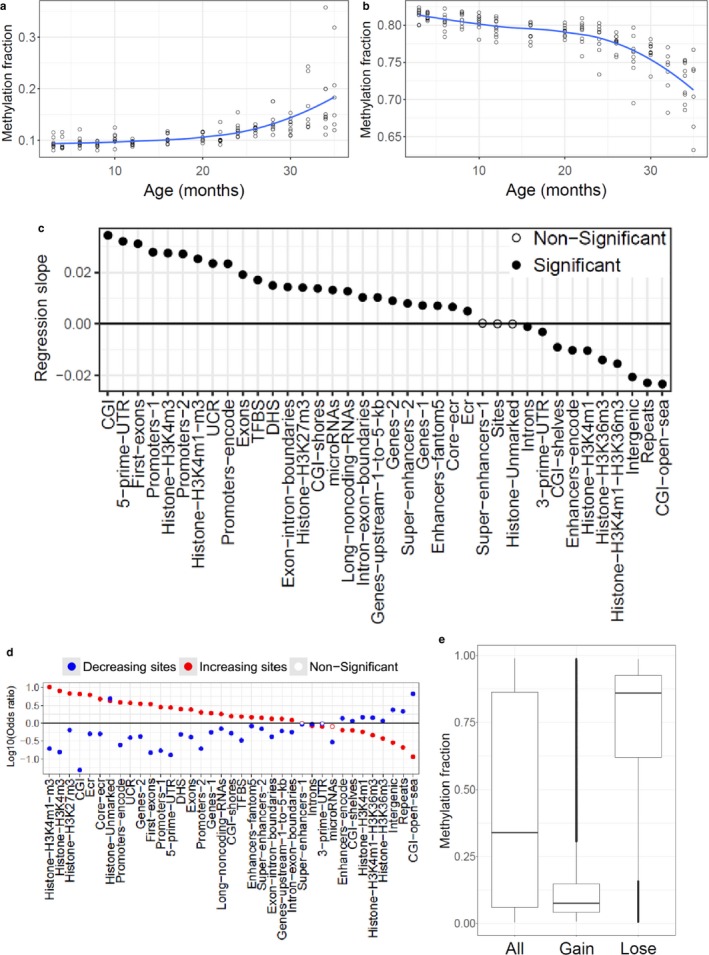
Global and region‐specific changes in DNA methylation with age. (a) Mean of the increasing and accelerating sites with the best model: $\text{age}∼{\text{methylation}}^{-\frac{1}{3}}$. (b) Mean of the decreasing and accelerating sites with the best model: $\text{age}∼{\text{methylation}}^{-\frac{1}{3}}$. (c) Linear regression slope of CpG sites that changed with age based on their assignment to indicated regions of the genome. (d) Enrichment analysis of significantly changing sites in indicated genomic regions. (e) Boxplot of the average methylation fraction across lifespan for all (All), gaining (Gain) and losing (Lose) methylation sites

Linear regression (fraction methylated *vs* age of animal) was further performed in a site‐by‐site manner, again accounting for possible confounding factors, and multiple data transformations were used to investigate nonlinear trends during aging (Table S1), with Akaike information criterion (AIC) indicating the best fitting models. We found that 21.2% of the sites were significantly correlated with age, including 10.2% that gained methylation and 11% that lost it (Figure S2a). Linear regression for significant sites revealed a slight decrease in methylation level (*p*‐value = 6.49 × 10^−17^; Figure S2b) with age. The best fitting model between significantly changing sites was age *vs* methylation percentage to the power of $-\frac{1}{3}$, observed in about 86% of the significant sites. These sites either gained methylation with age (41.6%; Figure 1a) or lost it (44.3%; Figure 1b) and changed predominantly in late life. In addition, 14% of the sites showed robust changes during early adulthood (Figure S3a,b). We similarly investigated the dataset of 651 human samples from the age of 19 to 101 years (Hannum et al., 2013), and the best fitting models were highly consistent with the mouse data ($\text{age}∼{\text{methylation}}^{-\frac{1}{3}}$: 81%, $\ln\left(\text{age}\right)∼{\text{methylation}}^{-\frac{1}{3}}$: 19% of the significant sites; Figures S4 and S5; Table S2).

We further separately examined the sites that gained and lost methylation with age (Figure 1e). Sites with increasing methylation were characterized by the low methylation status, and those with decreasing methylation by high methylation, compared with the average methylation levels (permutation test, *p*‐value < .0001 in both cases). The relationship between the regression slope and the mean methylation fraction for every site was also visualized (Figure S6) revealing a significant negative correlation (Pearson's correlation coefficient = −0.384, linear regression *p*‐value < 2 × 10^−16^). Again, sites with increasing methylation were hypomethylated, and those with decreasing methylation hypermethylated.

### 2.2 Various genomic regions show distinct remodeling patterns in DNA methylation

**Figure 2:**
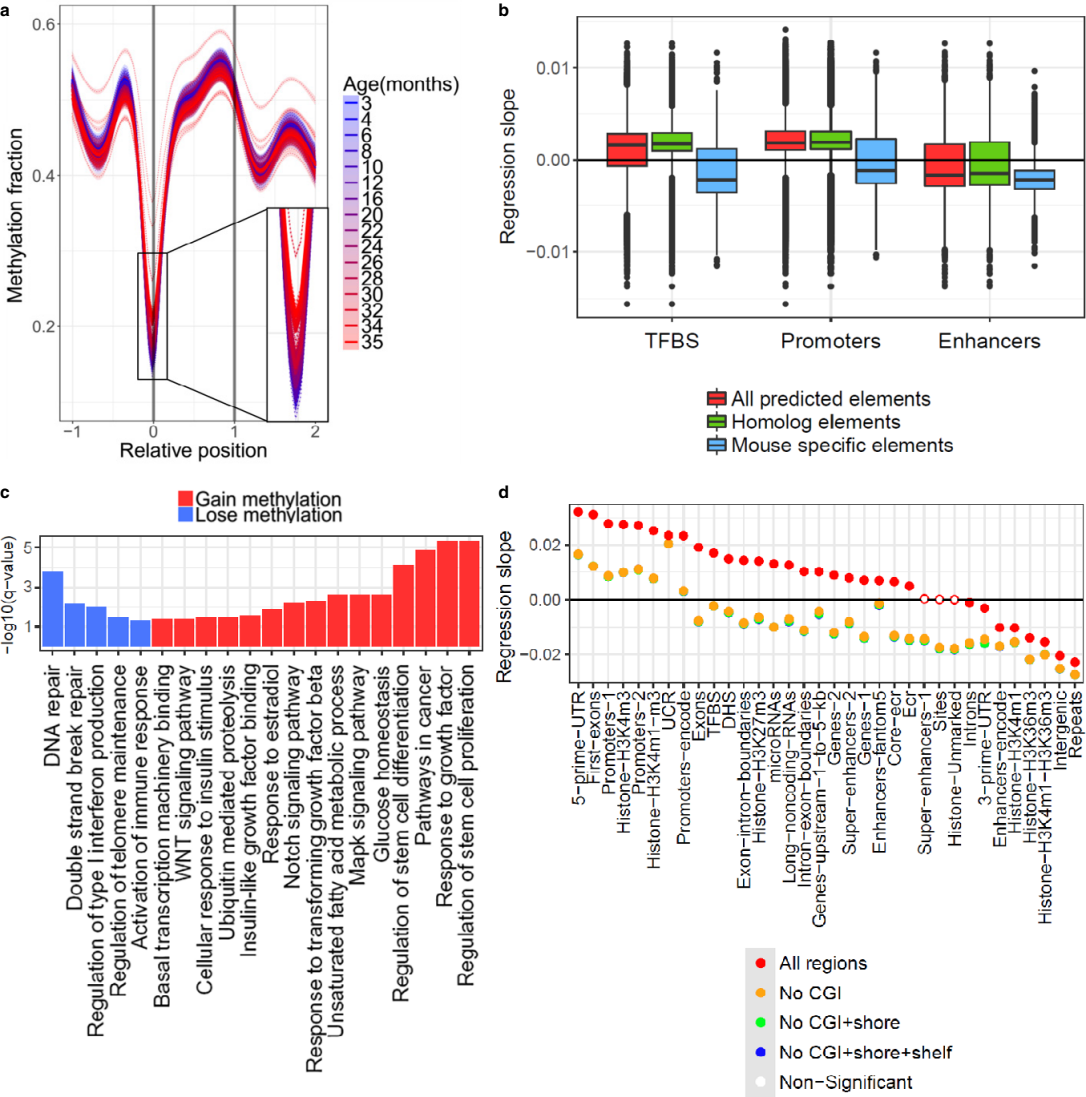
Genomic regions are characterized by distinct age‐related changes. (a) Age‐related changes in DNA methylation of genes. Relative position was calculated for every gene (0 corresponds to the TSS and 1 to the end of the gene) and extended in both directions up to the length of the gene. Dotted lines (individual samples) and thick lines (age groups) were calculated by generalized additive model using significant sites. (b) Regression slope of significantly changing sites in three genomic regions and differences between all sites (all predicted elements), human–mouse homologs (homolog elements), and mouse‐specific regions (mouse‐specific elements). (c) Significantly enriched pathways in promoters that gained methylation (red) and lost it (blue) during aging. KEGG and GO databases were used as pathway annotation for the analysis. (d) Linear regression slope of genomic regions after removal of significant sites that overlap with CpG islands (CGI), CGI shores, or CGI shelves

In addition to the global trends, we investigated changes in DNA methylation in distinct genomic regions. We performed linear regression analysis for various parts of the genome, following assignment of significant age‐related sites to annotated genomic regions (Figure 1c). The CpG methylation fraction was normalized by extracting the mean and dividing it by the standard deviation. Using this approach, we could examine dominant changes based on the number of changing sites, diminishing the effect of the degree of change for different sites that could potentially bias the results. We observed a strong age‐related gain in methylation in CpG islands (CGIs), 5′‐UTRs, first exons of genes and gene promoters; a strong increase in methylation could also be observed around the transcription start sites (TSSs; Figure 2a). We observed an age‐related methylation gain in many gene regions, such as exons and exon–intron boundaries, with the exception of introns and 3′‐UTRs, which tended to lose methylation with age. More broadly, increasing methylation was observed at the 5′‐ends and decreasing at the 3′‐ends of genes. Regulatory elements, such as microRNAs, long noncoding RNAs (Figure S7a), DNase hypersensitivity sites, and transcription factor binding sites (TFBS) tended to gain methylation during aging. We also observed increasing methylation in evolutionary conserved regions, core conserved regions, and ultra‐conserved regions. Regions with decreasing methylation included intergenic and repetitive regions (Figure S7b), the latter representing various classes of retrotransposons (Table S4). Overall, the mouse blood DNA methylome was characterized by robust remodeling with age, and its patterns and directions of change were dependent on the location of CpG sites within various functional regions of the genome.

We further investigated enrichment (Fisher exact test) of increasing and decreasing sites in different genomic regions (Figure 1d). Increasing sites tended to be enriched in regions that gained methylation and decreasing sites in regions that lost it with age. After correction for cell type composition changes during aging (Houseman et al., 2016), we found somewhat fewer significant sites (7%), revealing a pattern reminiscent of the original regional regression analysis (Figure S8). Finally, we compared changes during aging (regression slope) in promoters (Promoters‐Encode), enhancers (Enhancers‐Encode), and TFBS between all sites, sites in regions with human–mouse homology and sites in mouse‐specific regions (Figure 2b). Sites in homologous regions tended to gain, and sites in mouse‐specific groups to lose methylation during aging compared to all sites in that region and to each other (Student's *t* test, adjusted *p*‐value < .05).

### 2.3 Effects of CGIs, shores, and shelves on methylation trends

Consistent with previous studies (Florath, Butterbach, Muller, Bewerunge‐Hudler, & Brenner, 2014; Horvath et al., 2012; Rakyan et al., 2010), we found that the distance between the sites and CGIs strongly influenced methylation changes during aging, as CGIs gained and non‐islands lost methylation. CGIs may overlap with other genome regions, and the aging changes in them may be driven by overlapping islands and may be less characteristic of the region itself or non‐island parts of the genome. To examine this possibility, we removed, from every region, sites that could be assigned to (i) CGI, (ii) CGI and CGI shores, and (iii) CGI, CGI shore, and CGI shelves (Figure 2d). In most regions, CGIs had a strong effect on the overall pattern and mostly influenced regions that gained methylation. Many regions even changed their overall direction of change during aging after the removal of CGIs, but the most increasing regions, such as promoters, 5′UTRs and first exons, still gained methylation with age. Methylation changes during aging in ultra‐conserved regions tended to be less influenced by the overlapping CGIs than other regions that gained methylation. CGI island shores and shelves showed a minor additional effect.

### 2.4 Pathway enrichment of promoters and genes with age‐associated methylation changes

To get a deeper understanding of the possible biological impact of methylation changes during aging, we carried out pathway GSEA (Subramanian et al., 2005) on promoters (Table S5). We found 102 significantly enriched pathways (adjusted *p*‐value < .05) associated with the loss of methylation and 1,162 pathways associated with its gain in promoters during aging. Among the pathways with decreased methylation were those related to DNA repair, immune processes, and inflammation. In the increasing group, the most overrepresented pathways were related to developmental processes. There were also significantly enriched pathways related to aging and lifespan‐extending interventions, such as the response to growth factors, insulin‐like growth factor and TGFβ, MAPK cascade, WNT and Notch signaling pathway, regulation of stem cells, estradiol response, and fatty acid metabolism‐ and transcription regulation‐related pathways (Figure 2c; Carlson, Silva, & Conboy, 2008; Harrison et al., 2014; van Heemst, 2010; Heilbronn & Ravussin, 2003; Lopez‐Otin, Blasco, Partridge, Serrano, & Kroemer, 2013; Ott & Grune, 2014).

We also investigated the enrichment for genes (Table S6) and found 39 significant pathways that lost methylation and 987 pathways that gained it during aging. We observed similar patterns in gene bodies compared to promoters. Pathways with decreasing DNA methylation included DNA repair, immune function, and inflammation‐related pathways, and those with increasing methylation included various developmental pathways. There were aging‐related enriched pathways in the increasing group, including regulation of cell aging and senescence, growth factor response such as the response to TGFβ stimulus, stem cell proliferation and differentiation, MAPK cascade, WNT, Notch signaling, and fatty acid metabolism‐related pathways (Figure S9). In addition, a pathway involving DNA methylation itself was detected, including the gene *DNMT1*.

### 2.5 Regulation of the blood DNA methylome by calorie restriction

**Figure 3:**
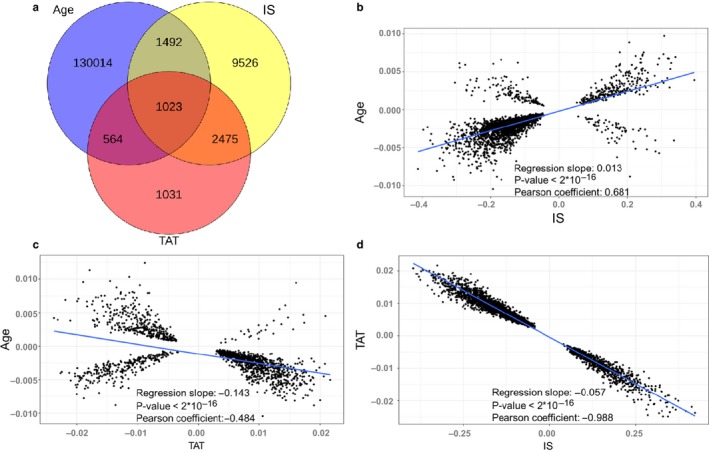
Age‐related DNA methylation changes that characterize calorie restriction (CR). (a) Venn diagram of CpG sites that significantly change with age in the control group (Age), sites with the initial shift (IS) in response CR, and sites with cumulative changes following the intervention (TAT). (b) Correlation between linear model coefficients of significant age‐related changes in the control group and significant IS changes. (c) Correlation between linear model coefficients of significant age‐related changes in the control group and significant cumulative changes under the CR regime. (d) Correlation between the coefficients of significant cumulative changes and significant IS changes in response to CR (including, but not limited to sites with age‐related changes)

We further investigated the effect of calorie restriction (CR), a classical lifespan‐extending intervention, on the blood DNA methylome by analyzing four groups of mice, from 10 till 27 months old, that were subjected to CR starting at the age of 4 months. We created a linear model and examined the sites characterized by the initial shift (IS) in response to CR (estimated shift at the age of 4 months) and the sites that changed with a different rate than the control group over time in response to treatment (designated time after treatment (TAT)), which represents the cumulative change (examples are in Figure S10a‐c). We detected 139,803 significant sites (22.6%; *F* test, adjusted *p*‐value < .05), where both IS and TAT had a significant effect. We then examined which effect, instantaneous or cumulative, may characterize CR. Our analysis revealed 5,093 sites that changed with TAT and 14,516 with IS (adjusted *p*‐value < .05). The identified sites were investigated in detail, revealing changes during (i) aging and IS, (ii) aging and TAT, and (iii) IS and TAT (Figure 3a).

First, we examined the relationship between the sites that changed significantly during aging in the control group and IS. There was a significant positive correlation between the linear model coefficients (Pearson's correlation coefficient = 0.681, *p*‐value < 2 × 10^−16^; Figure 3b). Thus, most sites with the initial shift following the intervention changed in the same direction as they did during aging. The same comparison between the sites changing both during aging and TAT showed a significant negative correlation (Pearson's correlation coefficient = −0.484, *p*‐value < 2 × 10^−16^; Figure 3c). The cumulative effect during CR, represented by the TAT, seemed to exhibit an opposite direction than the age‐related changes, and it slowed down the influence of aging on the methylome. We also investigated the sites, which change during both IS and TAT (including, but not limited to the sites with age‐related changes), and observed a strong negative correlation (Pearson's correlation coefficient = −0.988, *p*‐value < 2 × 10^−16^; Figure 3d). The data suggest that these sites initially change after the start of the intervention, but then the cumulative effect drives methylation back to the level of the control group. In addition, we examined 24 publicly available mouse samples (Cole et al., 2017; Hahn et al., 2017), which revealed similar trends in response to CR (Figure S11).

To get the end point of changes in the methylome in response to long‐term CR, we compared the oldest CR group (27 months) with the age‐matched control. For sites significantly changing during aging, we examined the relation between the regression slope throughout lifespan and the average difference in their methylation level between every control and CR site in the oldest age group (Figure S12a). We observed a significant negative correlation, consistent with the idea that long‐term CR slows down aging of the methylome. We further compared the average methylation difference between every control and CR site in the oldest age group and the age‐related regression slope of every site in all CR samples (four age groups) and observed a significant positive correlation (Figure S12b), showing that the long‐term cumulative changes shifted the methylome toward the direction of the cumulative effect itself. Based on these findings, we conclude that the initial and cumulative effects of CR show different effects and that the cumulative changes seem to slow down aging of the methylome during a long‐term treatment.

### 2.6 Effect of CR on mice in a different genetic background

To investigate whether our findings on CR apply to mice in a different genetic background, we investigated blood samples from 22 B6D2F1 male mice in two age groups. We detected 334 (0.048%) CpG sites differentially methylated between intervention and age‐matched control mice. We further investigated whether CR affected the same sites in a similar way in mice in different genetic backgrounds, that is, in B6D2F1 and C57BL/6 mice. For this, we examined the relation between the linear model coefficients of significant CR‐related changes in C57BL/6 mice and the same sites in B6D2F1 mice, assessing general relation of trends after CR in the two strains. Significant positive correlation was observed (Pearson's correlation coefficient = 0.295, *p*‐value < 2 × 10^−16^; Figure S13a). Then, we performed a similar analysis, but included only the sites, which showed significant CR‐related changes in both strains, by focusing on most reliable 204 sites. We again detected a significant positive correlation (Pearson's correlation coefficient = 0.969, *p*‐value < 2 × 10^−16^; Figure S13b). These data suggest that CR shifted the methylome in the same direction and generally affected the same sites in the two strains.

### 2.7 Increased entropy during aging is reflected in DNA methylation patterns

**Figure 4:**
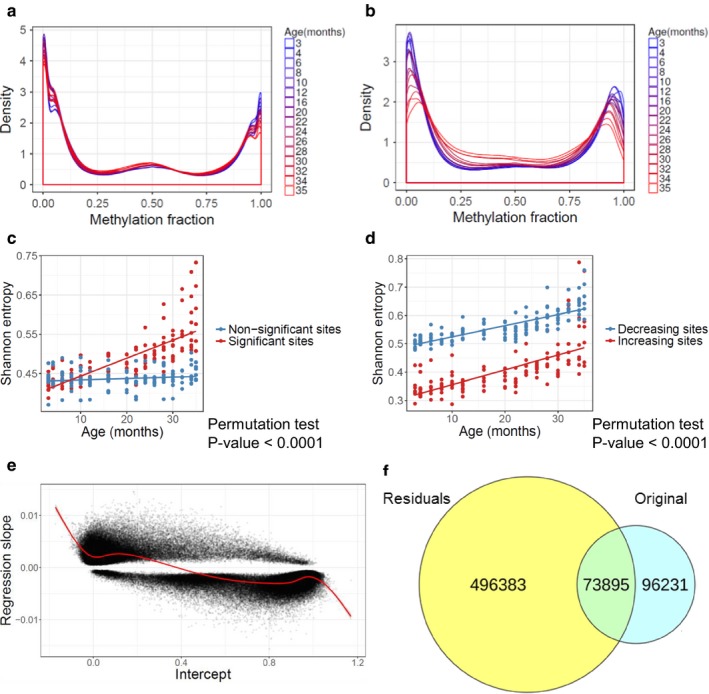
Age‐related changes in entropy of the DNA methylome. (a) Age‐related changes in DNA methylation, shown as a density plot accounting for all detected CpG sites. (b) Same as in (a), but the plots include only the CpG sites that significantly change with age. (c) Shannon entropy of the sites that significantly change (or do not change) with age. (d) Shannon entropy of the sites that significantly increase and decrease with age. (e) Intercept of the linear regression vs. the regression slope for every site that significantly changed with age. The red curve represents generalized additive model fit. (f) Venn diagram of significantly changed sites, based on the original age‐related changes (Original) and entropy‐normalized residuals (Residuals)

As discussed above, a characteristic feature of age‐related changes in DNA methylation was that the sites that gained methylation with age were initially hypomethylated, and those that lost it were hypermethylated. To further characterize age‐related methylation changes, we prepared density plots for every age group, including all sites (Figure 4a) or only the sites that showed significant changes with age (Figure 4b). Methylation levels for most sites were close to either 0 or 1; during aging, these extreme methylation states moved toward intermediate levels. These changes were even more pronounced among the sites that changed with age, pointing out to an age‐related increase in entropy. We quantified these changes by calculating Shannon entropy (Hannum et al., 2013) for individual samples and performing linear regression against age and confounders, verifying a significant increase in entropy with age. The rise in Shannon entropy was found for all sites (Pearson's correlation coefficient = 0.468, *p*‐value < 2 × 10^−16^; Figure S14a) and for the significant sites only (Pearson's correlation coefficient = 0.769, *p*‐value < 2 × 10^−16^; Figure S14b), the latter with the stronger effect (permutation test, *p*‐value < .0001; Figure 4c). We applied multiple data transformation to the age‐entropy dependence in the same way as described above and identified $\text{age}∼{\text{entropy}}^{-\frac{1}{3}}$ as the best fitting model based on AIC. We also found that entropy changed dominantly close to the end of life. Interestingly, there was a difference between entropy of the sites with increasing (Pearson's correlation coefficient = 0.682, *p*‐value < 2 × 10^−16^) and decreasing (Pearson's correlation coefficient = 0.801, *p*‐value < 2 × 10^−16^) methylation (Figure 4d): Entropy of the decreasing sites was higher for all age groups than of the sites with age‐related increased methylation (permutation test, *p*‐value < .0001). This could be seen in the density plots, wherein the peak with high methylation was wider than that with low methylation, and hypomethylated sites were more consistent in their methylation levels that the sites with high methylation.

In addition, an analysis of 651 human samples revealed increasing entropy with the best model: $\text{age}∼{\text{entropy}}^{-\frac{1}{3}}$, showing higher overall entropy of significant compared to nonsignificant sites and higher overall entropy of decreasing compared to increasing sites (Figures S15 and S16). Overall, increased entropy during aging was reflected in the methylation patterns and differentially affected the sites with increasing, decreasing, and unaffected methylation.

### 2.8 Normalization for the average entropy

To investigate sites, whose changes cannot be explained by the global change in entropy, we examined the relationship between the intercept of significant sites and the regression slope (Figure 4e) using a generalized additive model. Expected slope was then calculated for all sites, based on the intercept of linear regression and fitted values of generalized additive model, and the residual methylation fraction (RMF) was calculated by subtracting age × expected slope from the original methylation fractions (OMFs). After normalizing for the average entropy effect, linear regression was calculated for every RMF, accounting for confounding factors, using multiple data transformation, and choosing the best fitting model based on AIC. We found many sites (496,383 sites, 61.9%) without age‐related changes in OMF, but the RMF changed with age (Figure 4f). These sites had low or high methylation levels and resisted changes during aging. We observed 96,231 sites (12%), where OMF changed with age, but the entropy‐normalized RMF did not. Methylation of these sites changed during aging, but these changes could be explained by the average entropy effect. We also detected 73,895 (9.2%) sites that changed based on both OMF and RMF. These sites showed age‐related changes: 32.8% changed more than the average entropy, 49.5% less than the average entropy, and 17.7% changed in the opposite direction, that is, these sites were already highly methylated, but further increased methylation during aging and *vice versa*. Finally, 16.9% of the sites did not change in either analysis: These sites showed intermediate methylation levels and did not change with age. We conclude that most of the sites retain the early age methylation state, some follow or even exceed the average entropy trajectory, and few sites change in the direction against entropy.

Human data revealed a similar trend (Figure S17). We performed pathway enrichment analyses using the mouse samples and (i) sites that changed based on OMF and RMF, which showed age‐related changes that cannot be explained by the average entropy (Tables S7 and S8); and (ii) sites that changed based on just the OMF, which could be fully explained by the average entropy (Tables S9 and S10). We detected similar trends during this analysis; both average entropy‐driven and other sites were similarly enriched in pathways that changed during aging.

## 3 DISCUSSION

### 3.1 Global and local changes in DNA methylation throughout adult lifespan

Analysis of the blood DNA methylome of 141 mice representing 16 age groups spanning the entire adult lifespan (3‐35 months of age) allowed us to examine changes in DNA methylation in great detail and at a high resolution. The Robust changes were observed in late (86% sites) and early (14% sites) life, whereas few changes were seen throughout middle ages, suggesting that the primary changes during aging are not linear and that they either accelerate or decelerate with age in both mice and humans. Accelerating nature of the majority of CpG changes is consistent with the exponential increase in molecular damage and with the model of the rising deleteriome (Gladyshev, 2016), whereas developmental processes appear to underlie the decelerating sites during aging.

We observed strong methylation gain with age at the 5′ ends of genes. In contrast, loss of methylation occurred at the 3′UTRs and noncoding (introns) and intergenic regions. These findings in mice agree with previous human studies (McClay et al., 2014; Johansson et al., 2013), indicating conservation of age‐related changes in DNA methylation across species. We also observed increasing DNA methylation in other regulatory regions, such as microRNAs and long noncoding RNAs. CGI‐related methylation was previously shown to suppress the expression of microRNAs and contribute to human cancers (Lujambio & Esteller, 2009). These observations support interactions between different epigenetic mechanisms of aging. Repetitive regions showed decreasing methylation with age. These changes in retrotransposons may promote the expression of these elements and increase damage to the genome in late life (Robertson & Wolffe, 2000; Yoder, Walsh, & Bestor, 1997).

Evolutionarily conserved elements, most notably ultra‐conserved regions (100% sequence identity between human, mouse, and rat) gained methylation during aging. Increasing evidence suggests that these regions may be differentially expressed in human lymphomas and carcinomas (Calin et al., 2007). CGI hypermethylation suppresses the expression of the ultra‐conserved regions, which is not uncommon in human cancers (Lujambio et al., 2010). Human–mouse homologs seemed to be mostly gaining, whereas mouse‐specific regions losing methylation during aging. Overall, evolutionarily conserved elements tended to gain methylation during aging.

The presence of 5‐methylcytosine at CpG dinucleotides created a fivefold depletion of this sequence during vertebrate evolution, probably due to spontaneous and enzyme‐induced mutations (Bird, 1980; Gonzalgo & Jones, 1997). This may provide an explanation for hypomethylation of evolutionarily conserved regions and suggests that methylation gain during aging may promote mutations in the conserved elements in late life, contributing to the aging process.

CGIs were found to be among the regions that gained methylation most, similar to the results of human studies (Jones et al., 2015), and overlapping CGIs had a strong effect on the increasing regions. Regions with the strongest methylation gain, such as promoters, first exons, and 5′UTRs, showed a general methylation increase with age even when the overlapping CGIs were removed. Methylation gain in promoters was frequently associated with overlapping CGIs (Saxonov, Berg, & Brutlag, 2006), although our findings suggest that promoters even without overlapping CGIs gain methylation during aging.

Pathway enrichment analysis of promoters and genes illuminated the biology behind the changes. Genes and promoters that lost methylation tended to be enriched in pathways that maintain cell homeostasis, for example, DNA repair‐related pathways. These pathways are expected to become more expressed during aging, based on the idea of the inverse relationship between promoter methylation and gene expression (Jones et al., 2015). Many immunological and inflammation‐related pathways were also enriched, in agreement with the known increase in inflammation in late life (Lopez‐Otin et al., 2013). The most dominant pathways of genes and promoters gaining methylation during aging were developmental genes (Maegawa et al., 2017), which may indicate the decreasing expression of at least a subset of developmental genes during aging. There were also pathways in this group of promoters known to contribute to aging and lifespan‐extending interventions, such as regulation of cell response to growth factor stimulus (Lopez‐Otin et al., 2013), regulation of stem cell proliferation and differentiation (Heilbronn & Ravussin, 2003), insulin‐like growth factor and response to insulin‐related pathways (van Heemst, 2010), response to estradiol (Harrison et al., 2014), and ubiquitin‐mediated proteolysis (Ott & Grune, 2014). Promoters involved in fatty acid metabolism (Hahn et al., 2017), and WNT‐, Notch‐, and TGFβ‐related pathways also showed changes during aging (Carlson et al., 2008). Methylation changes in the MAPK cascade were reported previously during aging in mice (Hahn et al., 2017). There were pathways gaining methylation in gene bodies that were enriched for regulation of cellular aging and senescence. We also observed methylation‐related pathways enriched in gene bodies gaining methylation. Interestingly, a previous study showed decreased expression of *DNMT1* (Ray et al., 2006) in mouse T cells during aging; this is the main *DNMT* expressed in adulthood that maintains DNA methylation of adult dividing cells (Armstrong, Rakoczy, Rojanathammanee, & Brown‐Borg, 2014). These changes may contribute to misregulation of methylation machinery and a consequent entropy increase during aging. Cancer‐related pathways in promoters and gene bodies gained methylation, supporting the known relation between aging and cancer development (Lopez‐Otin et al., 2013). These enriched pathways indicate that DNA methylation may have a critical role during aging in mice.

### 3.2 Long‐term CR slows down aging of the methylome

CR is known to extend lifespan of mice and many other species (Bordone & Guarente, 2005). We could detect two distinct responses to this longevity intervention. First, our analyses revealed that, initially, CR shifted the DNA methylome in the same direction as aging. This initial shift may be at least partly a response to stress caused by CR. Second, the cumulative change during CR affected the methylation pattern differently (compared to aging and compared to the initial shift by CR), and this cumulative trend seemed to shift the methylome toward a younger state (compared to control) and/or slow down aging of the methylome.

Cumulative changes may influence the methylome in two different ways. On the one hand, they may affect sites with the initial shift caused by CR; in this case, the cumulative effect counteracts this shift. This effect may be compensatory with regard to the initial shift, as 98.2% of these sites do not differ from control in the oldest age group. On the other hand, the cumulative effect may influence the initially nonchanging sites and slow down the aging process. These sites appear to represent the long‐term, lifespan‐extending biological effect of CR. We observed that, initially, the methylome seemed to become older, whereas in the oldest age group the methylome appeared to be younger in the CR group, suggesting that the cumulative effect has a larger longevity impact following long‐term CR. The cumulative nature of CR is also confirmed by the meta‐analysis of longevity studies in mice, wherein aging rate, but not vulnerability parameter, was shown to be a crucial coefficient of the Gompertz model explaining the CR influence on lifespan (Garratt, Nakagawa, & Simons, 2016). Furthermore, the earlier start of the treatment strongly increases the degree of lifespan extension via CR in mice (Simons, Koch, & Verhulst, 2013).

Previous studies are consistent with our findings. Heterozygous *DNMT1* knockout mice are characterized by slower immune senescence (Richardson, 2003). Young *DNMT1* knockout mice feature general hypomethylation, the same trend as control mice during aging. On the other hand, older knockout mice showed hypermethylation and decreased immune senescence. Another study revealed that different sites change in response to CR in young and old mice (Hahn et al., 2017). A negative correlation was also shown between the methylation drift during aging and methylation changes following long‐term CR in mice and monkeys (Maegawa et al., 2017), and the severity and duration of CR may have influenced the resulting methylation patterns. Based on these findings, the cumulative effect of CR seems to slow down the aging pattern of the methylome and it may have a more important role for lifespan extension, compared to the transient initial shift. In addition, the role of the cumulative effect in our and other studies suggests a strong link between DNA methylation and aging. It should also be noted that the pattern of methylome changes following CR was similar in mice in different genetic backgrounds.

### 3.3 Increased entropy alters the developmental pattern

Most CpG sites changed with age toward intermediate methylation states, pointing to altered entropy of the methylome. Previous studies revealed increased entropy in human DNA methylation (Hannum et al., 2013) and in mouse livers aged 0.2 to 7.1 months old (Wang et al., 2017). We were able to examine changes in entropy across the whole mouse and human lifespan at a high resolution. Interestingly, we observed acceleration of entropy changes in older ages, in agreement with the rise in accumulating damage and the deleteriome (Gladyshev, 2016). Around 20% of the sites changed during aging, with the nearly equal numbers of those that increased and decreased methylation, but the global trend was associated with slightly decreased methylation with age, suggesting that the hypermethylated sites decrease somewhat more than the hypomethylated sites increase. Interestingly, however, hypomethylated sites that gained methylation were enriched in more pathways, including some with relevance to aging. The data suggest that these sites are more conserved and may underlie the features of aging associated with genetic programs, whereas the highly methylated sites that lost methylation with age may be associated with the more stochastic processes.

After normalization for the average entropy effect, we observed that many sites fully resisted changes (61.9%), defining the extremes in methylation levels. Some sites changed slower than the entropy changes (4.6%), other sites followed the average entropy effect (12%), but some changed even faster than the average entropy (3%). There were also few sites that changed in the direction opposite to entropy changes (1.6%). A recent study of human aging found that the majority of DNA methylation changes were associated with epigenetic drift and accumulating damage, supporting the disposable soma theory of aging, whereas targeted changes in methylation also exist, as predicted by the mutation accumulation theory, and may mediate the effects of aging‐related genes (Robins et al., 2017). Our results agree with these findings as the increasing and accelerating entropy, representing damage accumulation, disrupts the global methylation pattern, whereas fewer entropy‐independent targeted changes were observed in mouse and human samples, which may drive aging‐related biological functions. Interestingly, both entropy‐driven and nonentropy‐driven sites contributed similarly to the pathways changed during aging, indicating that the entropy growth of the methylome may have biological consequences. The early developmental program may define global and region‐specific methylation patterns and segregate functionally relevant CpG sites to fully methylated and unmethylated. This pattern may have multiple regulatory effects, with the respective sites contributing to certain biological functions. Other sites may follow entropy increase, which accelerates in late life and disrupt the developmental patterns.

Overall, we characterized changes in the blood DNA methylome of mice at unprecedented detail, uncovering nonlinear trends in DNA methylation remodeling during aging. Promoters and genes with significant age‐related changes in methylation turned out to be enriched in many known aging‐related pathways, pointing to an important link between DNA methylation and control of the aging process at the molecular level. In addition, analyses of CR revealed differences between the initial and cumulative effects of the intervention on the DNA methylome, the latter being important for lifespan extension. Analysis of DNA methylation allowed us to quantify entropy that both increased and accelerated with age and altered the developmental methylation patterns, even though many CpG sites resisted these changes. Finally, sites with low methylation levels were more conserved, and associated with biological functions relevant to aging. Taken together, our findings define the biological relevance of DNA methylation to aging.

## 4 EXPERIMENTAL PROCEDURES

### 4.1 Animals

Mice used in this study were obtained from the NIA Age Rodent Colony. Calorie restriction started at the age of 14 weeks and continued until the animals were sacrificed. For all other mice, food was provided ad libitum. Reduced representation bisulfite sequencing was performed using blood from the inferior vena cavae, and details can be found in Supplementary Experimental Procedures. The original data (Petkovich et al., 2017) were deposited to GEO under accession number GEO: GSE80672.

### 4.2 Data processing and statistical analysis

Downstream analyses and statistical analyses were performed using R. Methylation fraction was calculated by dividing the number of methylated reads by every read associated with a CpG. Nearly two million CpG sites (1,976,976 sites) were selected, which were present in all samples. We used a soft cutoff to exclude the sites with low coverage. Sites were excluded, which had <10× coverage in more than 50% of samples in any age group and in CR samples. Unlike microarray data, where all CpGs have certain variance, sites detected with RRBS can be either totally methylated or not methylated across all samples or in a group of samples (Sun, Cunningham, Slager, & Kocher, 2015). To decrease the effect of constant CpGs, we filtered out the sites with smaller standard deviation than the mean of the shortest interval that covers half of the standard deviations (Takeuchi, 1973). We used RnBeads R package to aggregate the measured sites, which belonged to the same CpG, to exclude high coverage outliers and sites that were overlapping with single nucleotide polymorphism. Detailed statistical methods and the genomic databases, used to annotate CpG sites, can be found in Supplementary Experimental Procedures.

## CONFLICT OF INTEREST

None declared.
