# Supplementary material for: Global remodeling of the mouse DNA methylome during aging and in response to calorie restriction
Source: Aging Cell. 2018 Mar 25;17(3):e12738. doi: 10.1111/acel.12738 (PMC5946071; doi:10.1111/acel.12738)
Supplement: Supplementary file 1 [file ACEL-17-e12738-s001.pdf]

Supplementary Information

Global remodeling of the mouse DNA methylome during aging and in response to calorie restriction

András Sziráki, Alexander Tyshkovskiy, and Vadim N. Gladyshev

Table of contents

<u>**Supplementary Experimental Procedures**</u>

<u>**Supplementary Figures:**</u>

1) **Supplementary Figure 1. Global state of the mouse blood DNA methylome.**
2) **Supplementary Figure 2. Age-related changes in the blood DNA methylome.**
3) **Supplementary figure 3. Sites with robust changes during early adulthood in mice.**
4) **Supplementary figure 4. Sites with robust changes during late adulthood in humans.**
5) **Supplementary figure 5. Sites with robust changes during early adulthood in humans.**
6) **Supplementary Figure 6. Relationship between the methylation state and the direction of change.**
7) **Supplementary Figure 7. Age-related changes in DNA methylation of certain genomic elements.**
8) **Supplementary Figure 8. Pathway gene set enrichment analysis of genes with changing methylation levels during aging.**
9) **Supplementary Figure 9. Correction for cell type heterogeneity.**
10) **Supplementary Figure 10. Examples of sites that change methylation with age following CR.**
11) **Supplementary Figure 11. Age-related DNA methylation changes that characterize calorie restriction in 24 publicly available mouse samples.**
12) **Supplementary Figure 12. Methylation state of samples following long-term calorie restriction.**
13) **Supplementary Figure 13. Analysis of calorie restriction in a different mouse strain.**
14) **Supplementary Figure 14. Age-related increases in entropy of the mouse DNA methylome.**
15) **Supplementary Figure 15. Age-related increases in entropy of the DNA methylome in human samples.**
16) **Supplementary Figure 16. Age-related changes in entropy of the DNA methylome in human samples.**
17) **Supplementary Figure 17. Normalization for average entropy across 651 human samples.**

<u>**Supplementary Tables:**</u>

1) **Supplementary Table 1. Data transformation and multiple models.**
2) **Supplementary Table 2. Data transformation and multiple models in human samples.**
3) **Supplementary Table 3. Detailed sample list** (separate file).
4) **Supplementary Table 4. Repetitive genomic regions in sites that increased and decreased methylation levels during aging** (separate file).
5) **Supplementary Table 5. Pathway GSEA of promoters changing methylation levels during aging** (separate file).
6) **Supplementary Table 6. Pathway GSEA of genes changing methylation levels during aging** (separate file).

7) **Supplementary Table 7. Functional enrichment for promoters associated with changes based on both the original and residual methylation fraction** (separate file).
8) **Supplementary Table 8. Functional enrichment for genes associated with changes based on both the original and residual methylation fraction** (separate file).
9) **Supplementary Table 9. Functional enrichment for promoters associated with changes based on just the original methylation fraction** (separate file).
10) **Supplementary Table 10. Functional enrichment for genes associated with changes based on just the original methylation fraction** (separate file).

Supplementary Experimental Procedures

Reduced representative bisulfite sequencing

Reduced Representation Bisulfite Sequencing (RRBS) of mouse blood samples was performed previously (Petkovich et al. 2017). Quality of high throughput sequence libraries was verified using "FastQC v.0.10.1" package (www.bioinformatics.babraham.ac.uk/projects/fastqc/). Trim Galore! v.0.4.0 was used for adapter removal and quality trimming. The TrimGalore tool (www.bioinformatics.babraham.ac.uk/projects/trim_galore/) was performed with settings optimized for RRBS. Methylation sites were detected using Bismark v.0.14.5 according to the program's manual.

Genomic databases

We used several databases to annotate CpG sites. RnBeads R package (Assenov et al. 2014) was employed to annotate genes (Genes-1) and promoters (Promoters-1), and the RnBeads package used Ensembl gene definitions and defined promoters as regions spanning 1,500 bases upstream and 500 bases downstream of the transcription start site (TSS) of the corresponding gene. We used annotatr R package (Cavalcante & Sartor 2017) to annotate genes (Genes-2; original database: UCSC genes), promoters (Promoters-2; 1 kb upstream of the TSS), CpG islands (CGIs), CGI shores (CGI-shores; 2 kb upstream/downstream from the ends of CGIs), CGI shelves (CGI-shelves, 2 kb upstream/downstream of the farthest upstream/downstream limits of CGI shores), the remaining CGI Open Sea genomic regions (CGI-open-sea), 1-5 kb upstream of the TSS (Genes-upstream-1-to-5-kb), 3'- and 5'-untranslated regions (3'UTRs, 5'UTRs), exons (Exons), first exons (First-exons), introns (Introns), intergenic regions (Intergenic), exon-intron and intron-exon boundaries (Exon-intron-boundaries, Intron-exon-boundaries; defined as 200 bp up/downstream of any boundary between an exon and intron), enhancers (Enhancers-fantom5; original database FANTOM5 (Andersson et al. 2014)), long non-coding RNA (Long-noncoding-RNAs; original database: GENCODE). We used the Mouse ENCODE Consortium (Yue et al. 2014) data to annotate seven different histone modifications (Histone-H3K27m3, Histone-H3K36m3, Histone-H3K4m1-m3, Histone-H3K4m1-H3K36m3, Histone-H3K4m1, Histone-H3K4m3, Histone-Unmarked; the database was merged from 15 different mouse tissues and cell types), DNase hypersensitive sites (DHS), predicted promoters (Promoters-encode), predicted enhancers (Enhancers-encode), transcription factor binding sites (TFBS) and the mouse specific and human-mouse homologs of the listed genomic regions.

We downloaded genomic repetitive regions (Repeats) using the UCSC Table Browser (Rosenbloom et al. 2015; Karolchik et al. 2004), and super-enhancers from the SEA database (Super-enhancers-1) (Wei et al. 2016) and the dbSUPER database (Super-enhancers-2) (Khan & Zhang 2016). Evolutionary conserved regions (Ecr) were defined as at least 100 bp long and 70% identity regions, and core evolutionary conserved regions (Core-ecr) were at least 350 bp and 77% identity regions between mouse and 10 different species (Human, Macaque, Chimp, Rat, Dog, Opossum, Chicken, Frog, Zebrafish, and Fugu). These regions were downloaded from

https://ecrbase.dcode.org (Loots & Ovcharenko 2007). Ultra conserved regions (UCR) were downloaded from http://ucbase.unimore.it (Lomonaco et al. 2014; Bejerano et al. 2004). Micro RNA (microRNAs) genomic coordinates were downloaded from http://www.mirbase.org (Kozomara & Griffiths-Jones 2014). Genomic coordinates were mapped to mm10 with UCSC Batch Coordinate Conversion (liftOver) tool (Hinrichs et al. 2006) when the original genome assembly was not mm10 in certain databases. Genomic coordinates of regions were filtered out; if we could not detect DNA methylation, we kept the coordinates of the regions, in which at least one methylated CpG could have been measured.

Statistical analysis

After data preprocessing and filtering, we used linear regression to examine the relationship between age and methylation levels and included 4 confounding factors: flow cell, library, adaptor and the number of aligned paired end reads of the samples. When site-by-site linear regressions and multiple data transformations were performed, we added the value 1 to every methylation fraction value to avoid incompatibility between the methylation value 0 and certain data transformations (Supplementary Table 1); we further chose the best fitting model based on the Akaike information criterion. We declared a site to be significant, if the p-value, adjusted by the Benjamini-Hochberg procedure, was smaller than 0.05. To avoid overfitting, we excluded significant sites, for which there was a direction change in the regression slope upon comparison of the linear regression with all our confounders and without the confounders. To determine the enrichment of increasing or decreasing (I/D) sites in certain genomic regions, we created a contingency table including the number of I/D sites in the genomic region, the number of I/D sites outside the region, the number of non-I/D sites in the region and the number of non-I/D sites outside the region, followed by a Fisher exact test.

To visualize the overall methylation change pattern in genes, we calculated relative positions for CpGs. The relative position 0 was assigned to the TSS, and 1 was the end of the gene. We also extended this analysis to the regions of the same length upstream and downstream of the gene and subjected them to the generalized additive model, using RnBeads R package, with a modification wherein we only included the significantly changing sites. To investigate the differences between the homolog elements, mouse-specific elements and all predicted elements, we performed Student's t-test between the regression slopes of every site in these regions and calculated adjusted p-values, using Benjamini-Hochberg procedure. When we examined the effect of the CpG islands, shores and shelves, we removed, from every region, the sites that could be assigned to (i) CGI, (ii) CGI and CGI shores, and (iii) CGI, CGI shore and CGI shelves, based on the built-in annotation of the RnBeads R package for these genomic regions, and performed a linear regression for every region using just the significantly changing sites. We included the four technical confounders, as described previously, and normalized every site by extracting the mean and dividing it by the standard deviation. In addition, we included, as confounders in every site, whether the site was present in a CGI shore, shelf or Open Sea region to avoid bias of the different aging trends.

Permutation tests were calculated by extracting the mean of the two compared groups, then merging the two groups into one. From the merged group, we randomly selected values for two groups (including the same number of values as the original groups). We extracted the means and

repeated the process 10,000 times. P-value was calculated by counting the number of cases, where the absolute value of the mean extraction of the randomly selected groups was larger than the absolute value of the original mean extraction, divided by 10,000. When testing the difference between increasing, decreasing and all sites, we aggregated the age groups by calculating the mean methylation across lifespan for every site.

Pathway enrichment analysis

To identify pathways associated with genes and promoters with changing methylation status during aging, we performed a pathway GSEA on a pre-ranked list of promoters and genes (Subramanian et al. 2005). This list included the sites annotated as promoters or gene bodies and z-scores, calculated as:

$$-ln(P) * S,$$

where P is the p-value of the linear regression of the site and S is the sign of the regression slope. For every promoter and gene, we calculated the mean of the z-scores of every site annotated to that genomic region. Kyoto Encyclopedia of Genes and Genomes (KEGG) and Gene Ontology (GO) Biological Process (BP) and Molecular Function (MF) databases were used in this analysis.

To identify pathways associated with age-related changes of methylation status, which can or cannot be explained by increasing entropy, we performed pathway enrichment analysis for promoters and genes that showed changes based on the original methylation fraction (OMF) or the residual methylation fraction (RMF) using Database for Annotation, Visualization and Integrated Discovery (DAVID) (Huang et al. 2009a; Huang et al. 2009b). Z-scores were calculated by using the equation (1) for sites showing increasing or decreasing changes based on (i) just the OMF; and (ii) both in OMF and RMF. Increasing or decreasing trends were determined by the changes based on the OMF. Average z-score was calculated for every site annotated to promoters or genes. We used the first 2,000 genomic regions with the highest absolute z-scores as an input for the DAVID analysis. We used KEGG, GO BP and MF databases with the threshold FDR = 0.05.

Analyses of calorie restriction

Investigating the effect of the calorie restriction (CR), we analyzed 20 C57BL/6 male mice in 4 age groups (10, 18, 23, 27 months). Using the original 141 C57BL/6 mice (16 age groups) as controls, we built a linear model including age, CR (0 for control and 1 for CR) and the time after treatment (TAT), which is the difference between the age of mouse and 4 months (i.e. the start of CR) in the case of the intervention group and 0 in control animals. Using this model, we could estimate the initial shift (IS) at the beginning of the CR treatment (4 months) and calculate the difference in the rate of changes during aging in the CR group versus the control group, using the TAT which represents the cumulative change. We performed a site-by-site linear regression including the above mentioned variables and used a partial F-test to decide if IS and the TAT had a significant effect together (Benjamini-Hochberg FDR = 0.05). Partial F-test was calculated by fitting the reduced (including only the age) and the full (including the age, CR and TAT) models separately and thereafter comparing them using Analysis of variance (ANOVA). Then we used the

significant sites based on the F-test and determined, for every site, whether IS and TAT had a significant effect separately (Benjamini-Hochberg FDR = 0.05). To investigate the relation between the age-, initial shift- and cumulative effect-related changes we performed a linear regression between the linear model coefficients of these features.

We also investigated DNA methylation of blood samples from 22 (10 control and 12 CR) B6D2F1 male mice. Two age groups were analyzed in both subsets (20 and 27 months in controls, 21 and 27 months in CR). We applied a linear model and included age, intervention and flow cell as technical confounders. We compared the CR-related changes between the B6D2F1 and the C57BL/6 strain. To be able to make this comparison, we recalculated the C57BL/6 analysis, including only age and CR, but not TAT. Comparison between the two strains was by a linear regression between the CR-related linear model coefficients of the strains.

Analysis of publicly available data

We downloaded publicly available human and mouse data for comparative analyses of our findings. The dataset of 656 human whole blood samples (Hannum et al. 2013) was downloaded from NCBI's Gene Expression Omnibus: GSE40279. We performed multidimensional scaling and 5 samples were identified as outlier samples and excluded from further analyses. Site-by-site linear regressions and multiple data transformations were performed as detailed above and four confounding factors were included in the linear regressions: gender, ethnicity, source and plate information. Entropy analysis was performed on the human data as detailed above for mouse samples. We downloaded 24 mouse liver methylation data (Cole et al. 2017; Hahn et al. 2017) with accession numbers GSE89275 and GSE92486. We used the sites that were present in all samples and had higher coverage than 5x in more than 50% of samples in any age group and in calorie restriction samples. Furthermore, we used RnBeads R package to aggregate the measured sites, which belonged to the same CpG, to exclude high coverage outliers and sites that were overlapping with single nucleotide polymorphism. We built a linear model, including age (2, 5, 22, 26 months), calorie restriction (0 for control and 1 for CR), time after treatment and the source of the sample. To investigate the relationship between the age-, initial shift- and cumulative effect-related changes we performed linear regression between the linear model coefficients of these features.

References

Andersson R, Gebhard C, Miguel-Escalada I, Hoof I, Bornholdt J, Boyd M, Chen Y, Zhao X, Schmidl C, Suzuki T, Ntini E, Arner E, Valen E, Li K, Schwarzfischer L, Glatz D, Raithel J, Lilje B, Rapin N, Bagger FO, Jorgensen M, Andersen PR, Bertin N, Rackham O, Burroughs AM, Baillie JK, Ishizu Y, Shimizu Y, Furuhata E, Maeda S, Negishi Y, Mungall CJ, Meehan TF, Lassmann T, Itoh M, Kawaji H, Kondo N, Kawai J, Lennartsson A, Daub CO, Heutink P, Hume DA, Jensen TH, Suzuki H, Hayashizaki Y, Muller F, Consortium TF, Forrest ARR, Carninci P, Rehli M & Sandelin A (2014) An atlas of active enhancers across human cell types and tissues. *Nature* 507, 455–461.

Assenov Y, Muller F, Lutsik P, Walter J, Lengauer T & Bock C (2014) Comprehensive analysis of DNA methylation data with RnBeads. *Nat Meth* 11, 1138–1140.

Bejerano G, Pheasant M, Makunin I, Stephen S, Kent WJ, Mattick JS & Haussler D (2004) Ultraconserved elements in the human genome. *Science* 304, 1321–1325.

Cavalcante RG & Sartor MA (2017) annotatr: Genomic regions in context. *Bioinformatics*.

Cole JJ, Robertson NA, Rather MI, Thomson JP, McBryan T, Sproul D, Wang T, Brock C, Clark W, Ideker T, Meehan RR, Miller RA, Brown-Borg HM & Adams PD (2017) Diverse interventions that extend mouse lifespan suppress shared age-associated epigenetic changes at critical gene regulatory regions. *Genome Biol.* 18, 58.

Hahn O, Gronke S, Stubbs TM, Ficz G, Hendrich O, Krueger F, Andrews S, Zhang Q, Wakelam MJ, Beyer A, Reik W & Partridge L (2017) Dietary restriction protects from age-associated DNA methylation and induces epigenetic reprogramming of lipid metabolism. *Genome Biol.* 18, 56.

Hannum G, Guinney J, Zhao L, Zhang L, Hughes G, Sadda S, Klotzle B, Bibikova M, Fan J-B, Gao Y, Deconde R, Chen M, Rajapakse I, Friend S, Ideker T & Zhang K (2013) Genome-wide methylation profiles reveal quantitative views of human aging rates. *Mol. Cell* 49, 359–367.

Hinrichs AS, Karolchik D, Baertsch R, Barber GP, Bejerano G, Clawson H, Diekhans M, Furey TS, Harte RA, Hsu F, Hillman-Jackson J, Kuhn RM, Pedersen JS, Pohl A, Raney BJ, Rosenbloom KR, Siepel A, Smith KE, Sugnet CW, Sultan-Qurraie A, Thomas DJ, Trumbower H, Weber RJ, Weirauch M, Zweig AS, Haussler D & Kent WJ (2006) The UCSC Genome Browser Database: update 2006. *Nucleic Acids Res.* 34, D590-8.

Huang DW, Sherman BT & Lempicki RA (2009a) Bioinformatics enrichment tools: paths toward the comprehensive functional analysis of large gene lists. *Nucleic Acids Res.* 37, 1–13.

Huang DW, Sherman BT & Lempicki RA (2009b) Systematic and integrative analysis of large gene lists using DAVID bioinformatics resources. *Nat. Protoc.* 4, 44–57.

Karolchik D, Hinrichs AS, Furey TS, Roskin KM, Sugnet CW, Haussler D & Kent WJ (2004) The UCSC Table Browser data retrieval tool. *Nucleic Acids Res.* 32, D493-6.

Khan A & Zhang X (2016) dbSUPER: a database of super-enhancers in mouse and human genome. *Nucleic Acids Res.* 44, D164-71.

Kozomara A & Griffiths-Jones S (2014) miRBase: annotating high confidence microRNAs using deep sequencing data. *Nucleic Acids Res.* 42, D68-73.

Lomonaco V, Martoglia R, Mandreoli F, Anderlucci L, Emmett W, Bicciato S & Taccioli C (2014) UCbase 2.0: ultraconserved sequences database (2014 update). *Database (Oxford).* 2014.

Loots G & Ovcharenko I (2007) ECRbase: database of evolutionary conserved regions, promoters, and transcription factor binding sites in vertebrate genomes. *Bioinformatics* 23, 122–124.

Petkovich DA, Podolskiy DI, Lobanov A V, Lee S-G, Miller RA & Gladyshev VN (2017) Using DNA Methylation Profiling to Evaluate Biological Age and Longevity Interventions. *Cell Metab.* 25, 954–960.e6.

Rosenbloom KR, Armstrong J, Barber GP, Casper J, Clawson H, Diekhans M, Dreszer TR, Fujita PA, Guruvadoo L, Haeussler M, Harte RA, Heitner S, Hickey G, Hinrichs AS, Hubley R, Karolchik D, Learned K, Lee BT, Li CH, Miga KH, Nguyen N, Paten B, Raney BJ, Smit AFA, Speir ML, Zweig AS, Haussler D, Kuhn RM & Kent WJ (2015) The UCSC Genome Browser database: 2015 update. *Nucleic Acids Res.* 43, D670-81.

Subramanian A, Tamayo P, Mootha VK, Mukherjee S, Ebert BL, Gillette MA, Paulovich A, Pomeroy SL, Golub TR, Lander ES & Mesirov JP (2005) Gene set enrichment analysis: a knowledge-based approach for interpreting genome-wide expression profiles. *Proc. Natl. Acad. Sci. U. S. A.* 102, 15545–15550.

Wei Y, Zhang S, Shang S, Zhang B, Li S, Wang X, Wang F, Su J, Wu Q, Liu H & Zhang Y (2016) SEA: a super-enhancer archive. *Nucleic Acids Res.* 44, D172-9.

Yue F, Cheng Y, Breschi A, Vierstra J, Wu W, Ryba T, Sandstrom R, Ma Z, Davis C, Pope BD, Shen Y, Pervouchine DD, Djebali S, Thurman RE, Kaul R, Rynes E, Kirilusha A, Marinov GK, Williams BA, Trout D, Amrhein H, Fisher-Aylor K, Antoshechkin I, DeSalvo G, See L-H, Fastuca M, Drenkow J, Zaleski C, Dobin A, Prieto P, Lagarde J, Bussotti G, Tanzer A, Denas O, Li K, Bender MA, Zhang M, Byron R, Groudine MT, McCleary D, Pham L, Ye Z, Kuan S, Edsall L, Wu Y-C, Rasmussen MD, Bansal MS, Kellis M, Keller CA, Morrissey CS, Mishra T, Jain D, Dogan N, Harris RS, Cayting P, Kawli T, Boyle AP, Euskirchen G, Kundaje A, Lin S, Lin Y, Jansen C, Malladi VS, Cline MS, Erickson DT, Kirkup VM, Learned K, Sloan CA, Rosenbloom KR, Lacerda de Sousa B, Beal K, Pignatelli M, Flicek P, Lian J, Kahveci T, Lee D, Kent WJ, Ramalho Santos M, Herrero J, Notredame C, Johnson A, Vong S, Lee K, Bates D, Neri F, Diegel M, Canfield T, Sabo PJ, Wilken MS, Reh TA, Giste E, Shafer A, Kutyavin T, Haugen E, Dunn D, Reynolds AP, Neph S, Humbert R, Hansen RS, De Bruijn M, Selleri L, Rudensky A, Josefowicz S, Samstein R, Eichler EE, Orkin SH, Levasseur D, Papayannopoulou T, Chang K-H, Skoultchi A, Gosh S, Disteche C, Treuting P, Wang Y, Weiss MJ, Blobel GA, Cao X, Zhong S, Wang T, Good PJ, Lowdon RF, Adams LB, Zhou X-Q, Pazin MJ, Feingold EA, Wold B, Taylor J, Mortazavi A, Weissman SM, Stamatoyannopoulos JA, Snyder MP, Guigo R, Gingeras TR, Gilbert DM, Hardison RC, Beer MA & Ren B (2014) A comparative encyclopedia of DNA elements in the mouse genome. *Nature* 515, 355–364.

Supplementary Figures

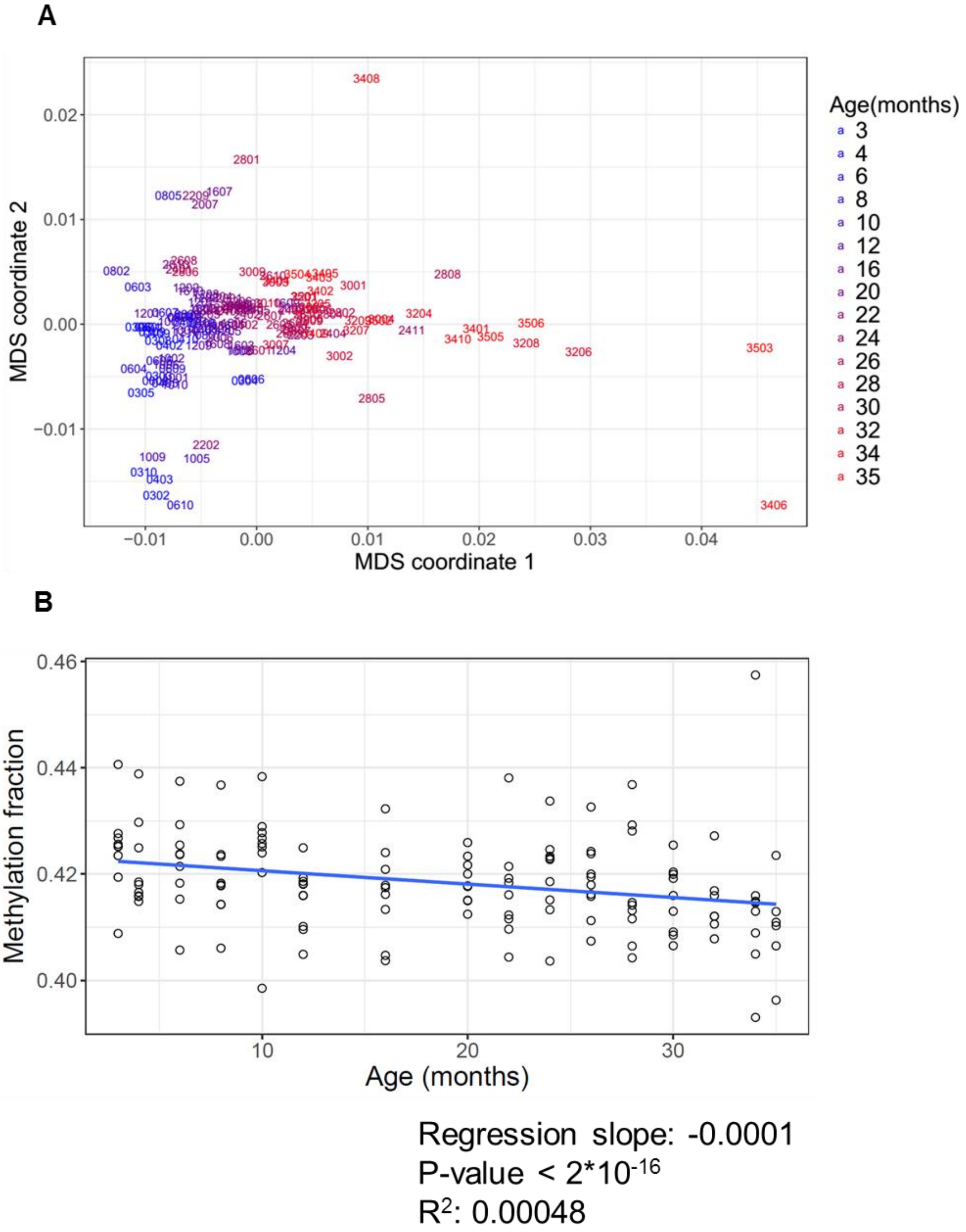

Supplementary Figure 1. Global state of the mouse blood DNA methylome. (A) Multi-dimensional scaling (MDS) of 141 C57Bl/6 male samples. Colors represent ages as shown on the right. Samples are represented by sample ID, wherein the first two numbers represent age and the second two represent identification number in an age group. **(B)** Mean methylation fraction of every site. Linear regression parameters were calculated by using every site.

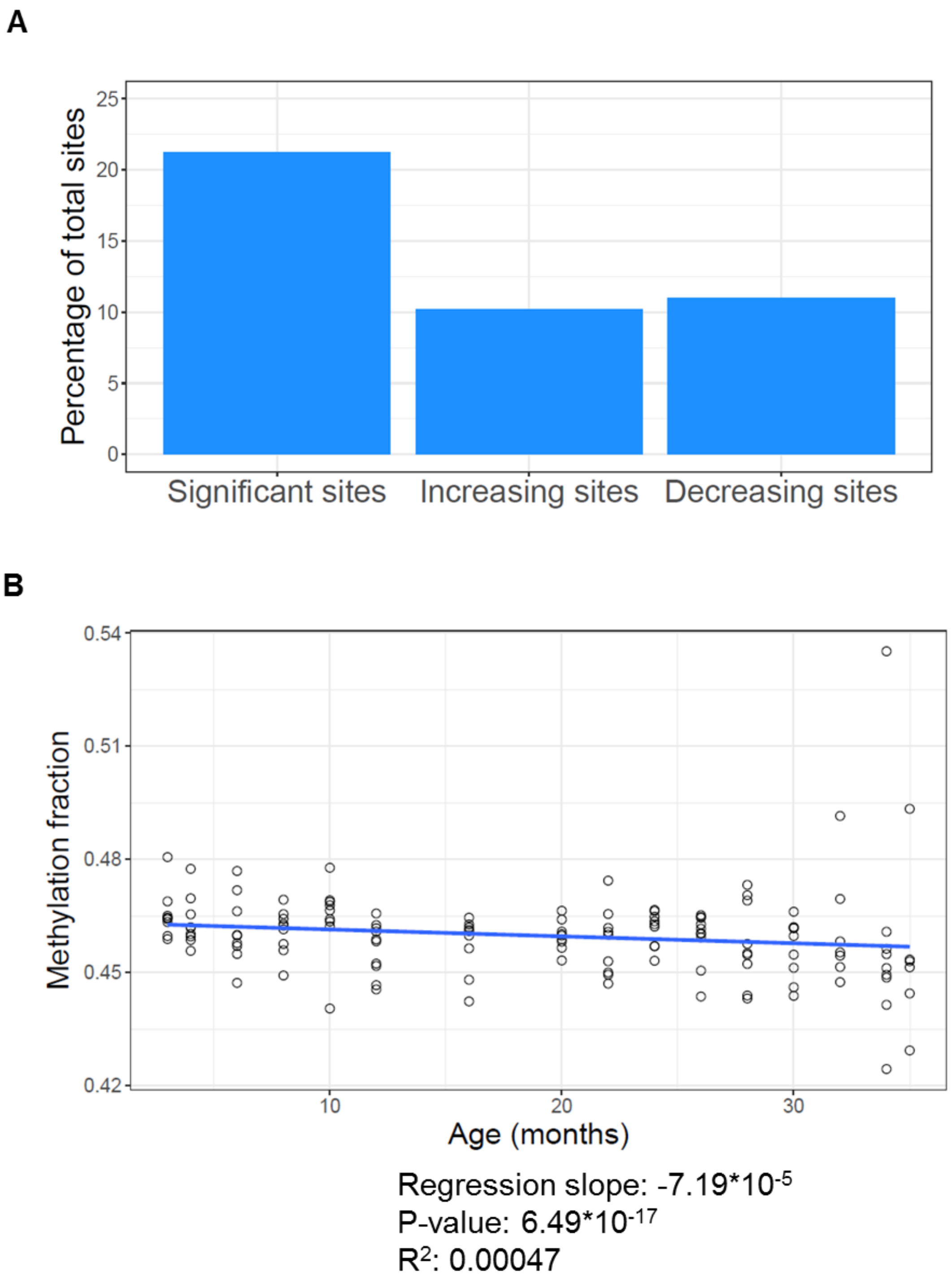

Supplementary Figure 2. Age-related changes in the mouse blood DNA methylome. (A) Percentage of CpG sites that significantly changed methylation levels, increased methylation and decreased methylation with age. **(B)** Mean methylation fraction of significantly changing sites. Regression parameters were calculated using every CpG site that changed with age.

A

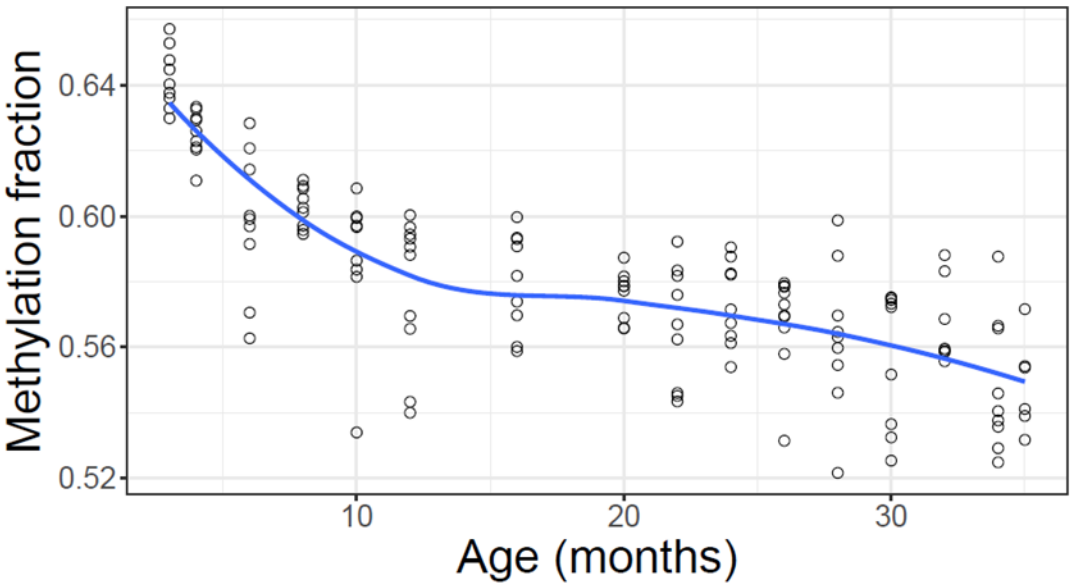

B

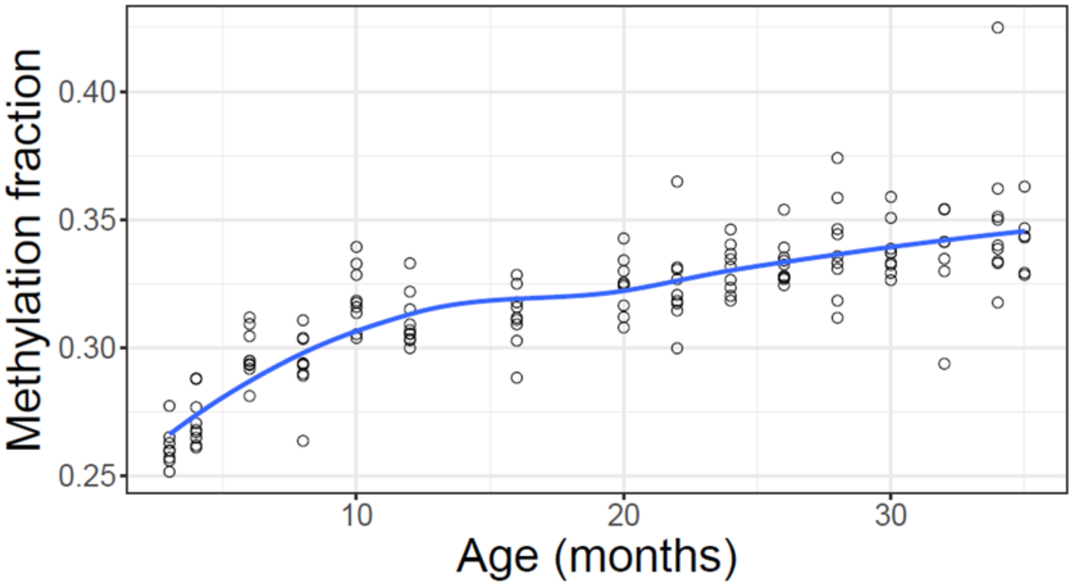

Supplementary figure 3. Sites with robust changes during early adulthood in mice. (A) Mean of significantly decreasing sites with the best model: $ln(age) \sim methylation^{-\frac{1}{3}}$. **(B)** Mean of significantly increasing sites with the best model: $ln(age) \sim methylation^{-\frac{1}{3}}$.

A

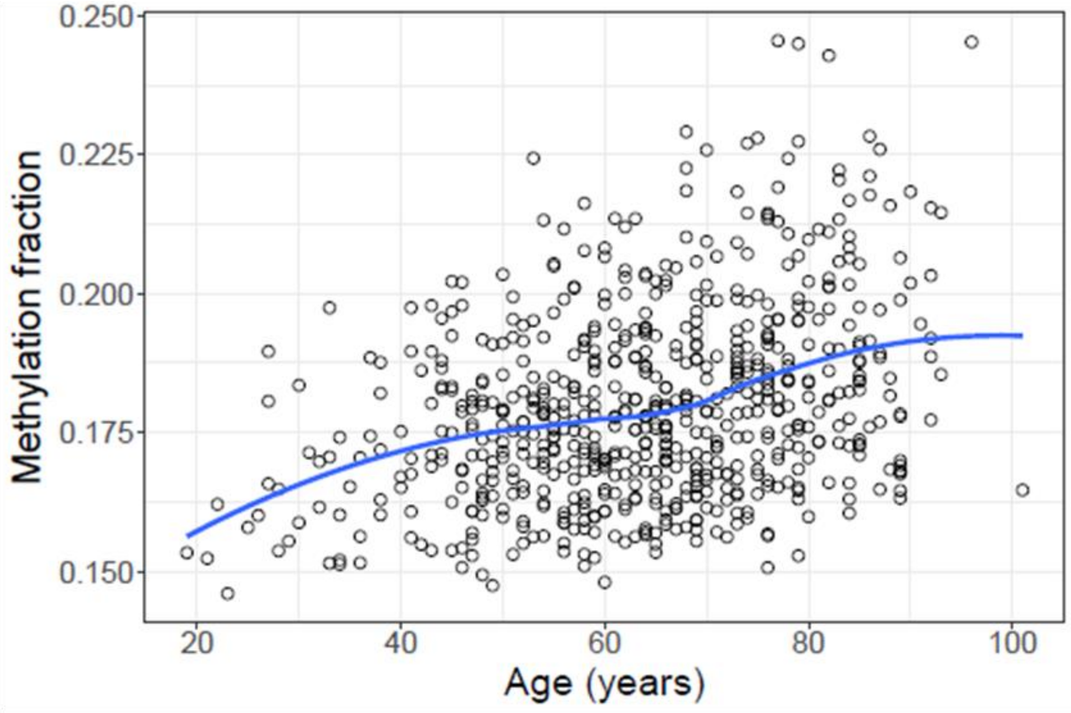

B

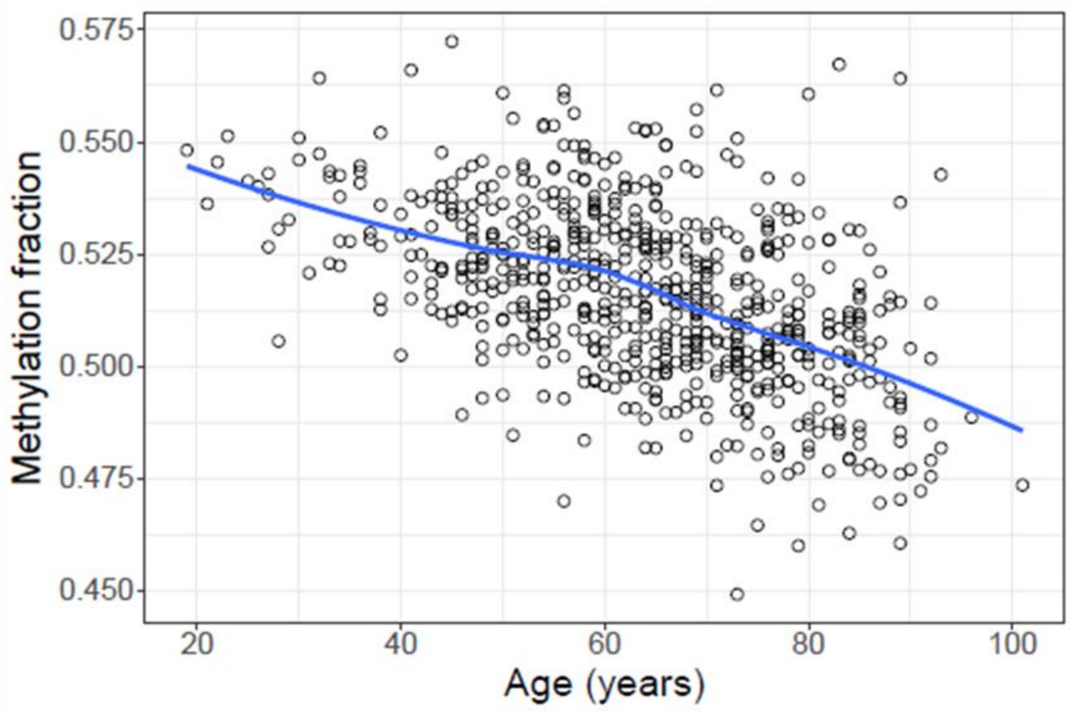

Supplementary figure 4. Sites with robust changes during late adulthood in humans. (A) Mean of significantly increasing sites with the best model: $age \sim methylation^{-\frac{1}{3}}$ in 651 human samples from the age of 19 to 101 years. **(B)** Mean of significantly decreasing sites with the best model: $age \sim methylation^{-\frac{1}{3}}$ in 651 human samples from the age of 19 to 101 years.

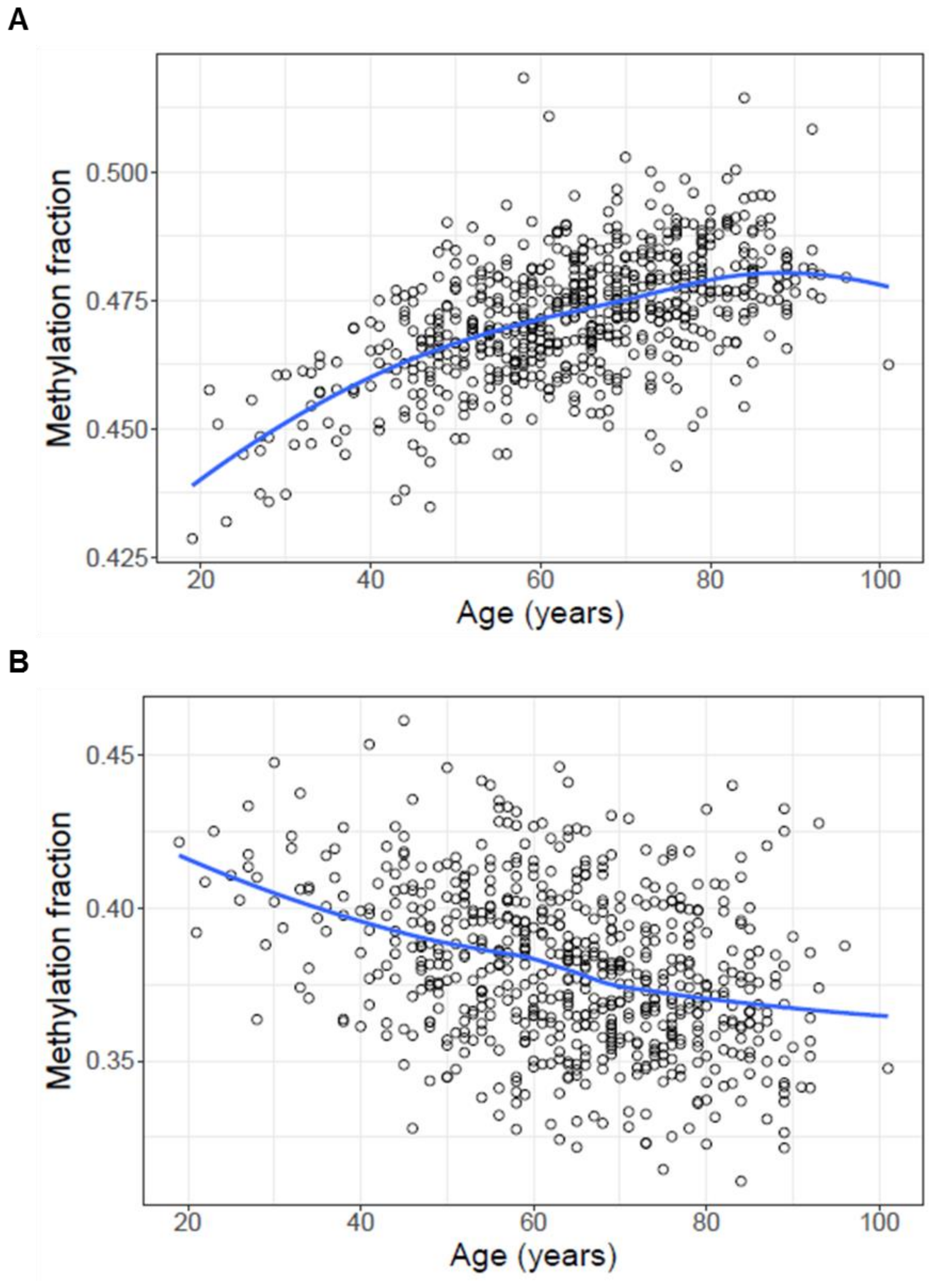

Supplementary figure 5. Sites with robust changes during early adulthood in humans. (A) Mean of significantly increasing sites with the best model: $ln(age) \sim methylation^{-\frac{1}{3}}$ in 651 human samples from the age of 19 to 101 years. **(B)** Mean of significantly decreasing sites with the best model: $ln(age) \sim methylation^{-\frac{1}{3}}$ in 651 human samples from the age of 19 to 101 years.

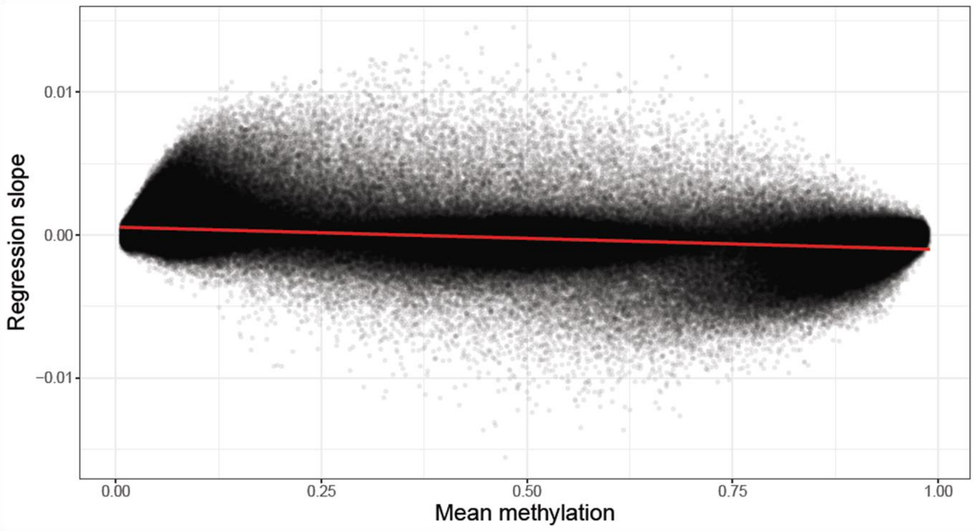

Regression slope: -0.0016
P-value < 2*10^{-16}
Pearson coefficient: -0.384

Supplementary Figure 6. Relationship between the methylation state and the direction of change. Correlation and linear regression between the linear regression slope and mean methylation fraction for every site.

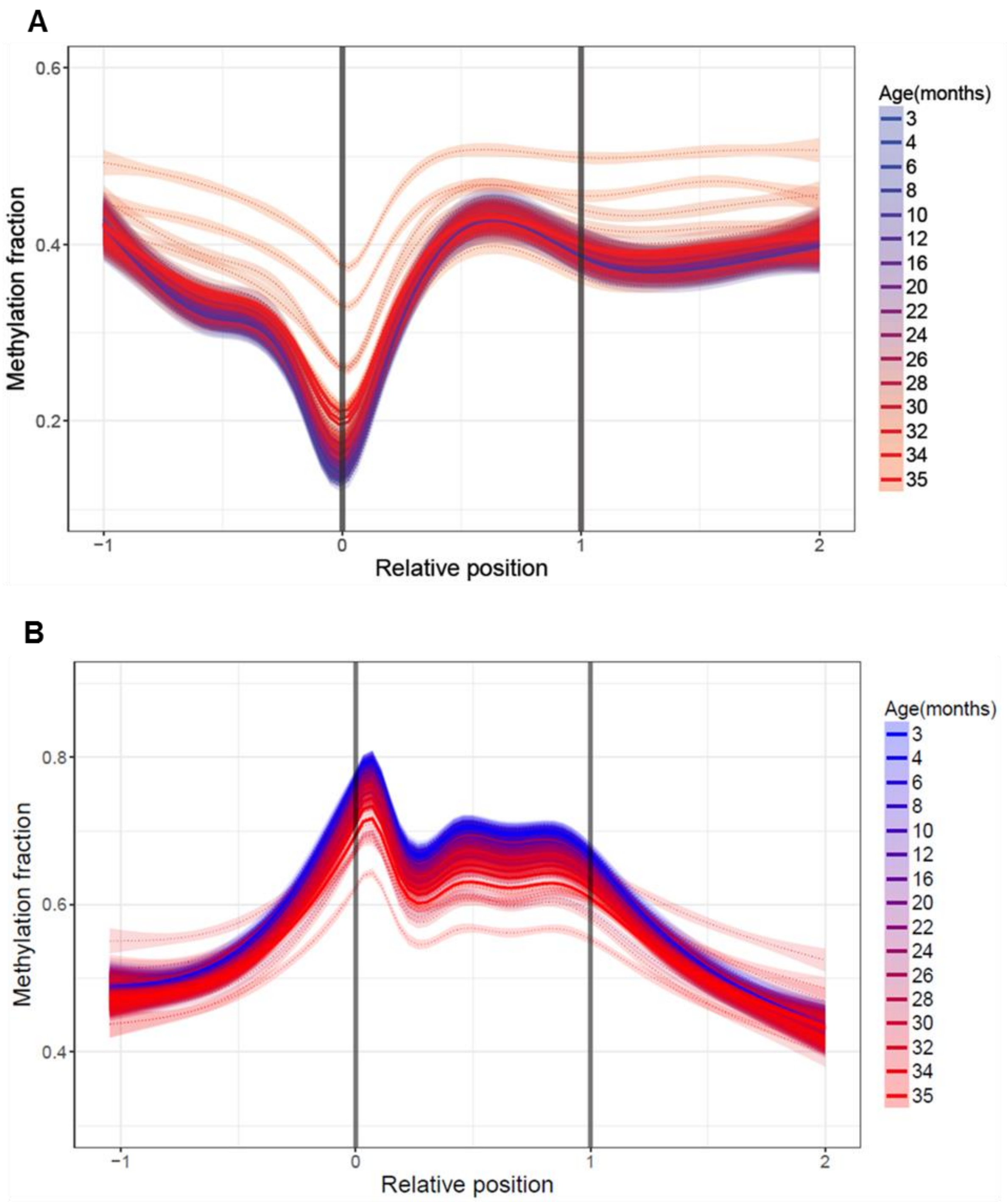

Supplementary Figure 7. Age-related changes in DNA methylation of certain genomic elements. (A) Age-related changes in methylation of long non-coding RNAs. **(B)** Age-related changes in methylation of repetitive elements in the genome. Relative positions are shown as 0 (where the element begins) and 1 (where the element ends) and extended upstream (-1) and downstream (2) according to the length of the genomic region. Lines were calculated by generalized additive model based on the significantly changed sites. Color scheme shows the age, dotted lines show individual samples, and thick lines show age groups.

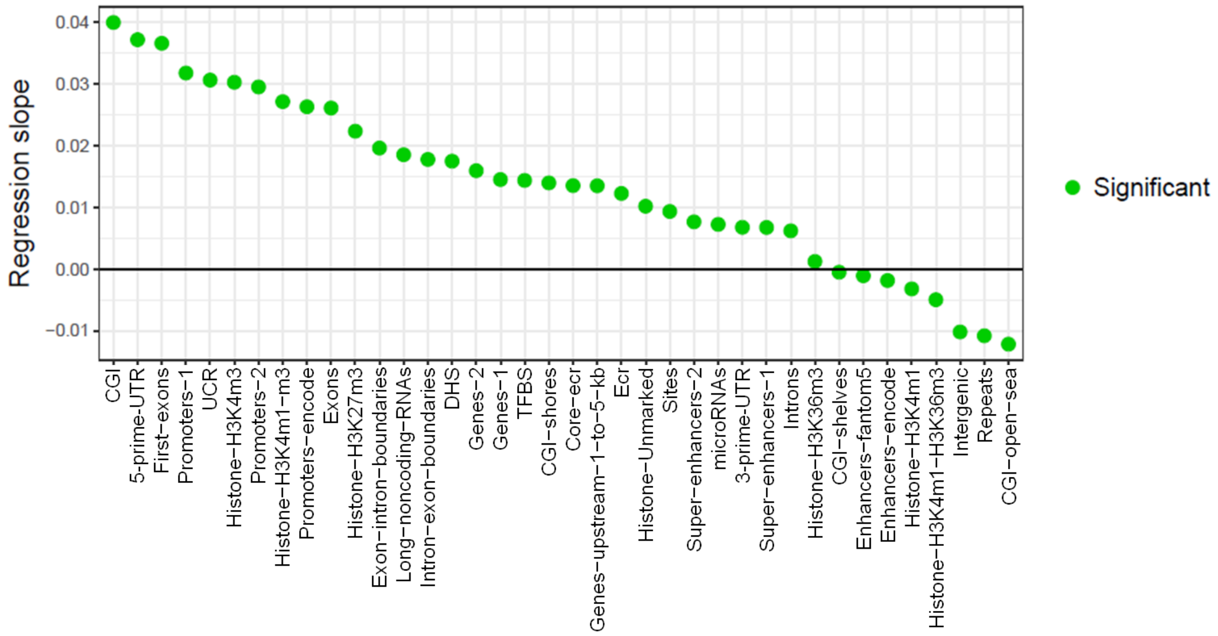

Supplementary Figure 8. Correction for cell type heterogeneity. Regression slope of linear regression for every genomic region, including only the significant sites, based on the RefFreeEWAS analysis.

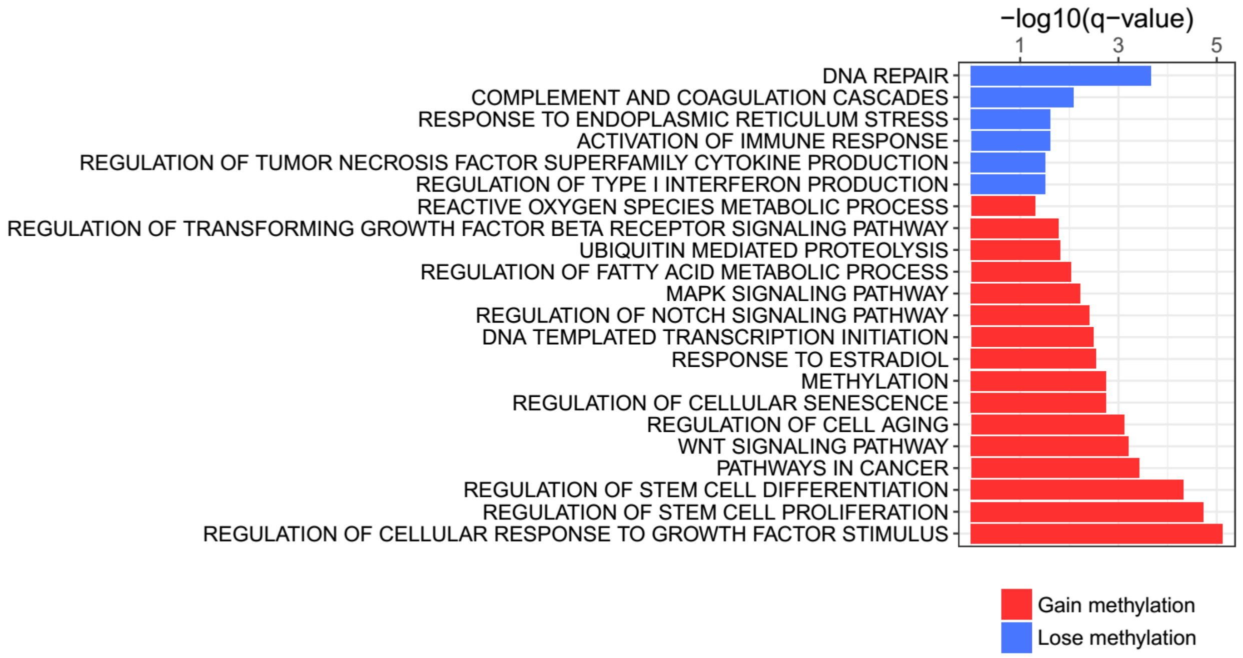

Supplementary Figure 9. Pathway gene set enrichment analysis of genes with changing methylation levels during aging. Examples of significantly enriched pathways in gene bodies. Pathways enriched by hypomethylated genes are colored in blue, and pathways enriched by hypermethylated genes are colored in red. KEGG and GO databases were used as a pathway annotation for the analysis.

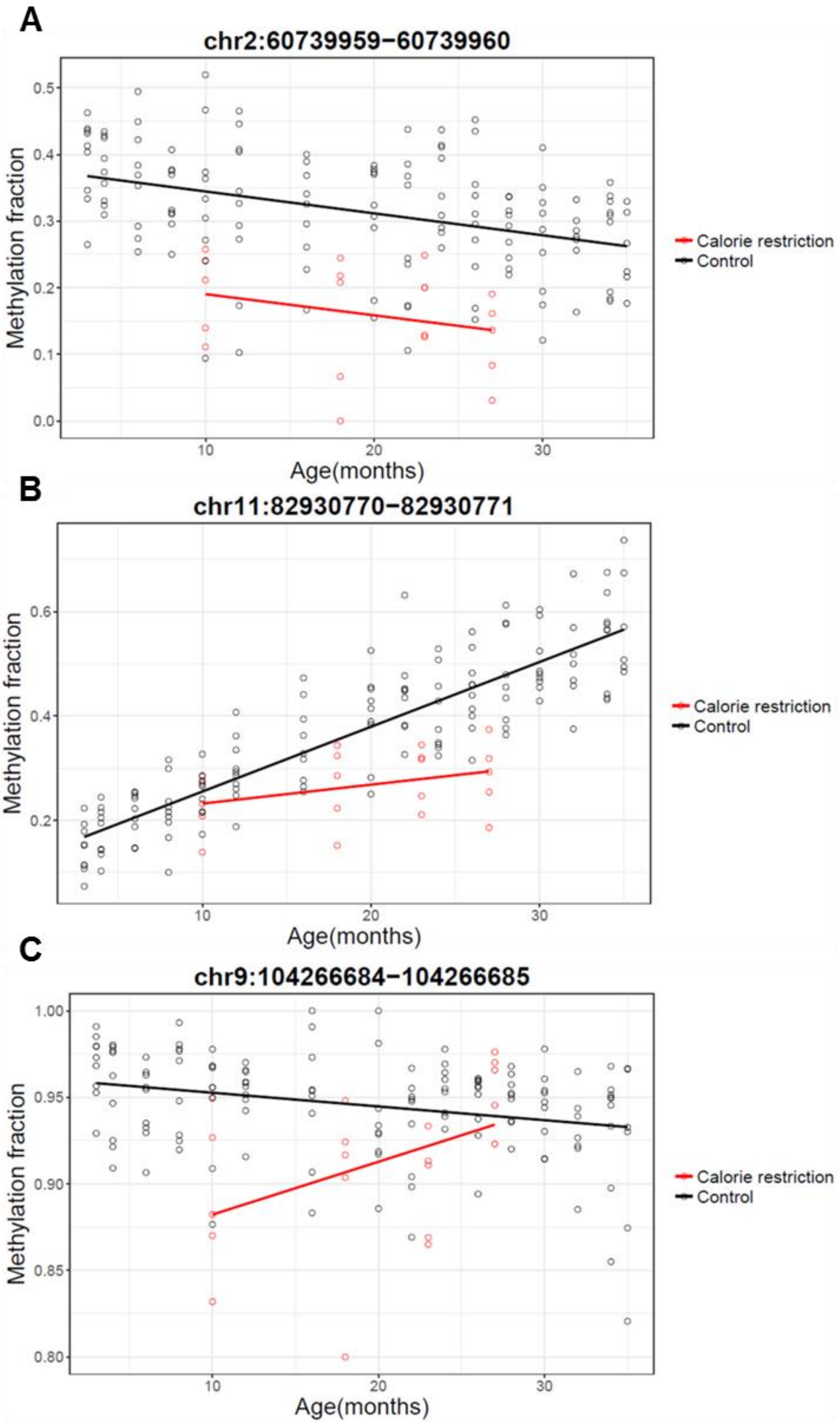

Supplementary Figure 10. Examples of sites that change methylation with age following CR. **(A)** Site with the initial shift, but no significant cumulative effect. **(B)** Site with the cumulative effect, but without a significant initial shift. **(C)** Site with the significant initial shift and also cumulative effect. In each case, the control group has a significant age-related change.

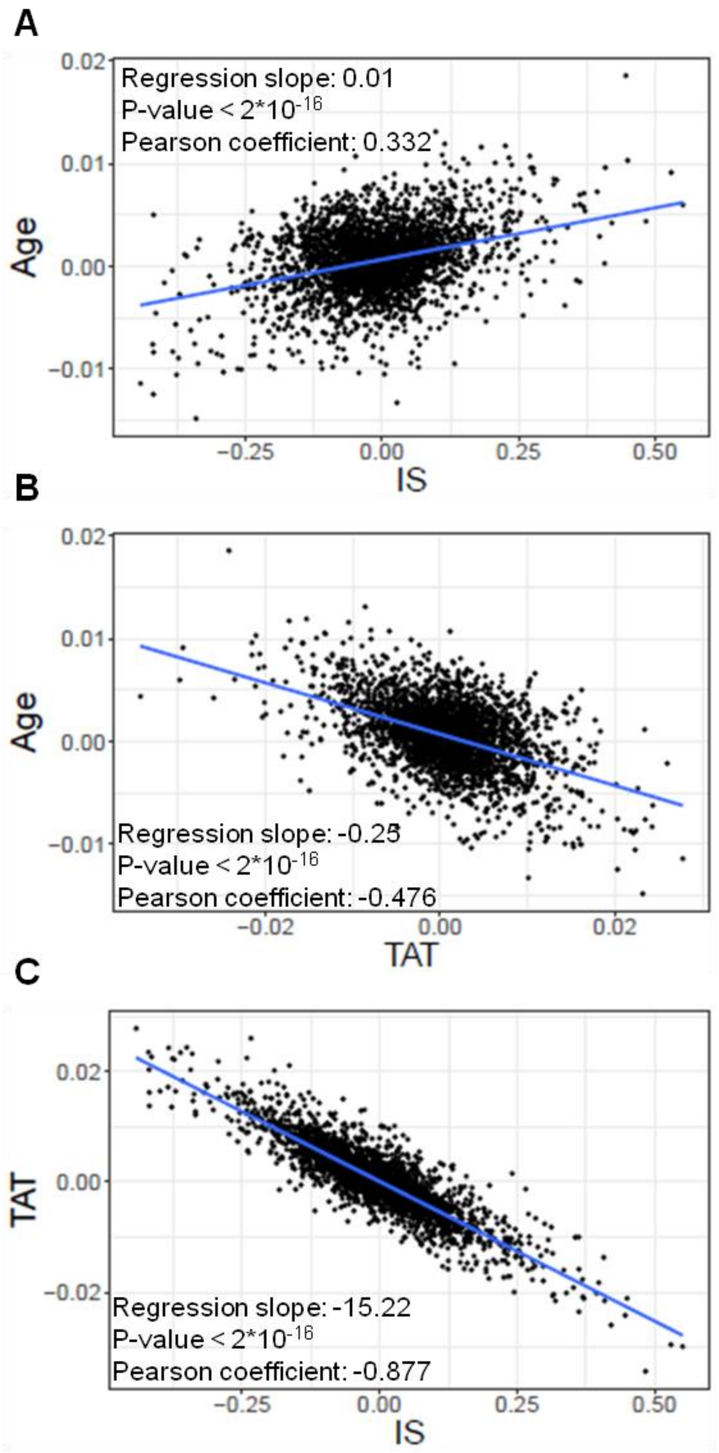

Supplementary Figure 11. Age-related DNA methylation changes that characterize calorie restriction in 24 publicly available mouse samples. (A) Correlation between linear model coefficients of age-related changes and the initial shift (IS). **(B)** Correlation between the linear model coefficients of age-related changes and cumulative changes under the CR regime (TAT). **(C)** Correlation between coefficients of cumulative changes and IS changes in response to CR.

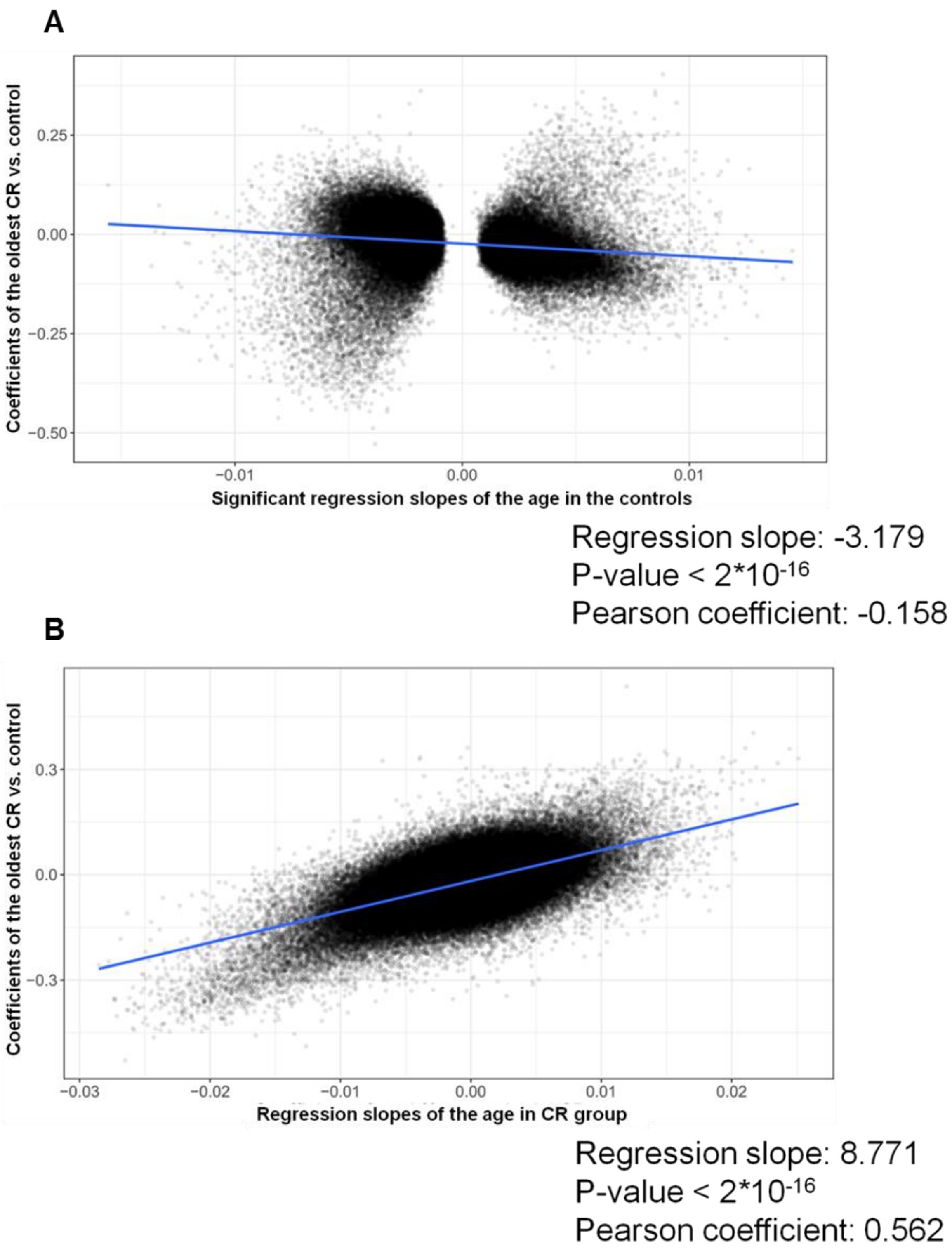

Supplementary Figure 12. Methylation state of samples following long-term calorie restriction. (A) Correlation between linear model coefficients of oldest (27 months) calorie restriction (CR) samples versus control samples and the regression slope of the significant age-related changes in the control group. **(B)** Correlation between linear model coefficients of the oldest (27 months) calorie restriction samples versus control samples and the regression slope of age-related changes in the calorie restriction samples.

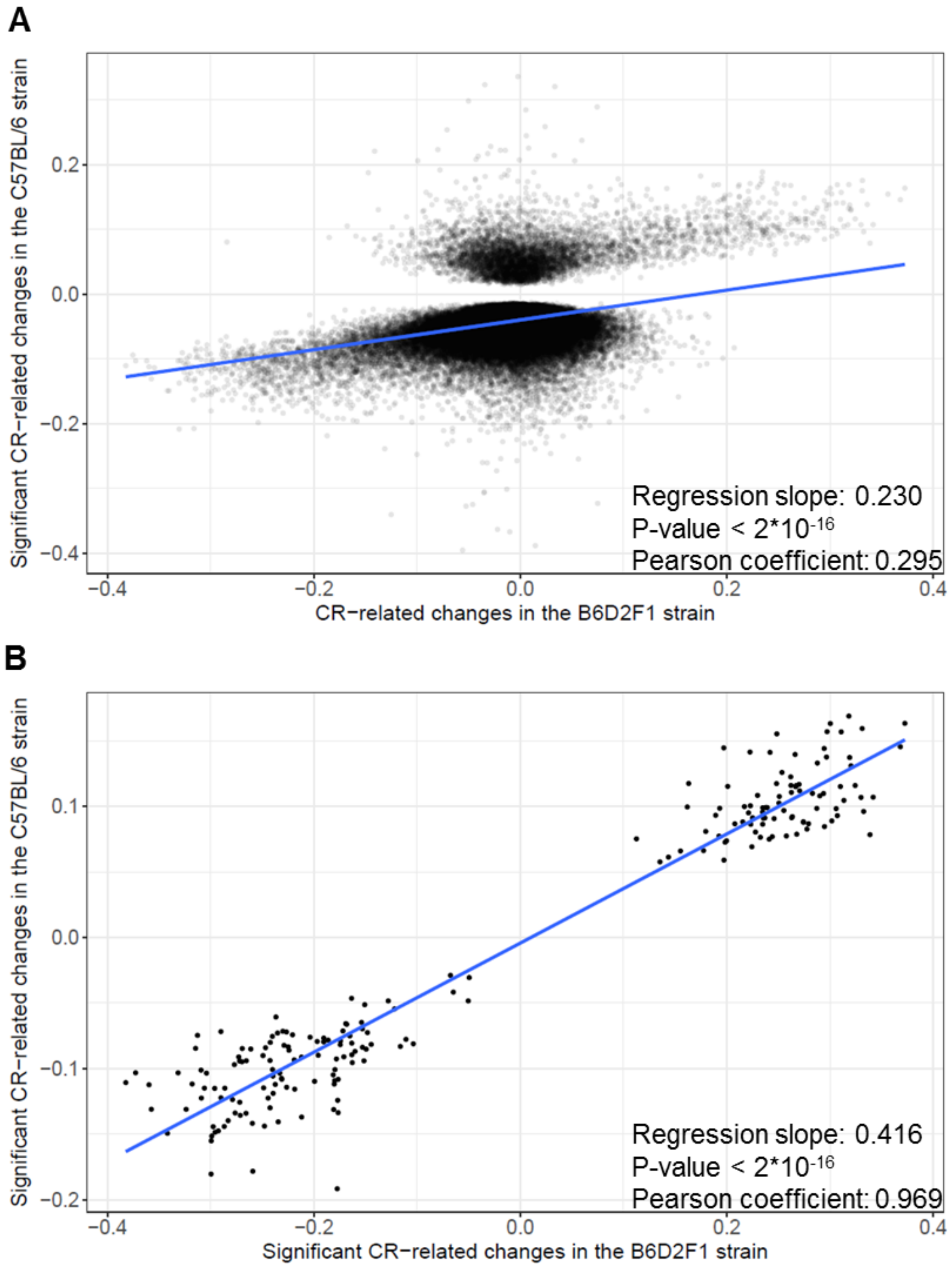

Supplementary Figure 13. Analysis of calorie restriction in a different mouse strain. (A) Correlation between the linear model coefficients of CR-related changes based on the significant sites in the C57BL/6 mice and the same sites in the B6D2F1 strain. **(B)** Correlation between the linear model coefficients of CR-related changes based on sites that are significant in both C57BL/6 and B6D2F1 strains.

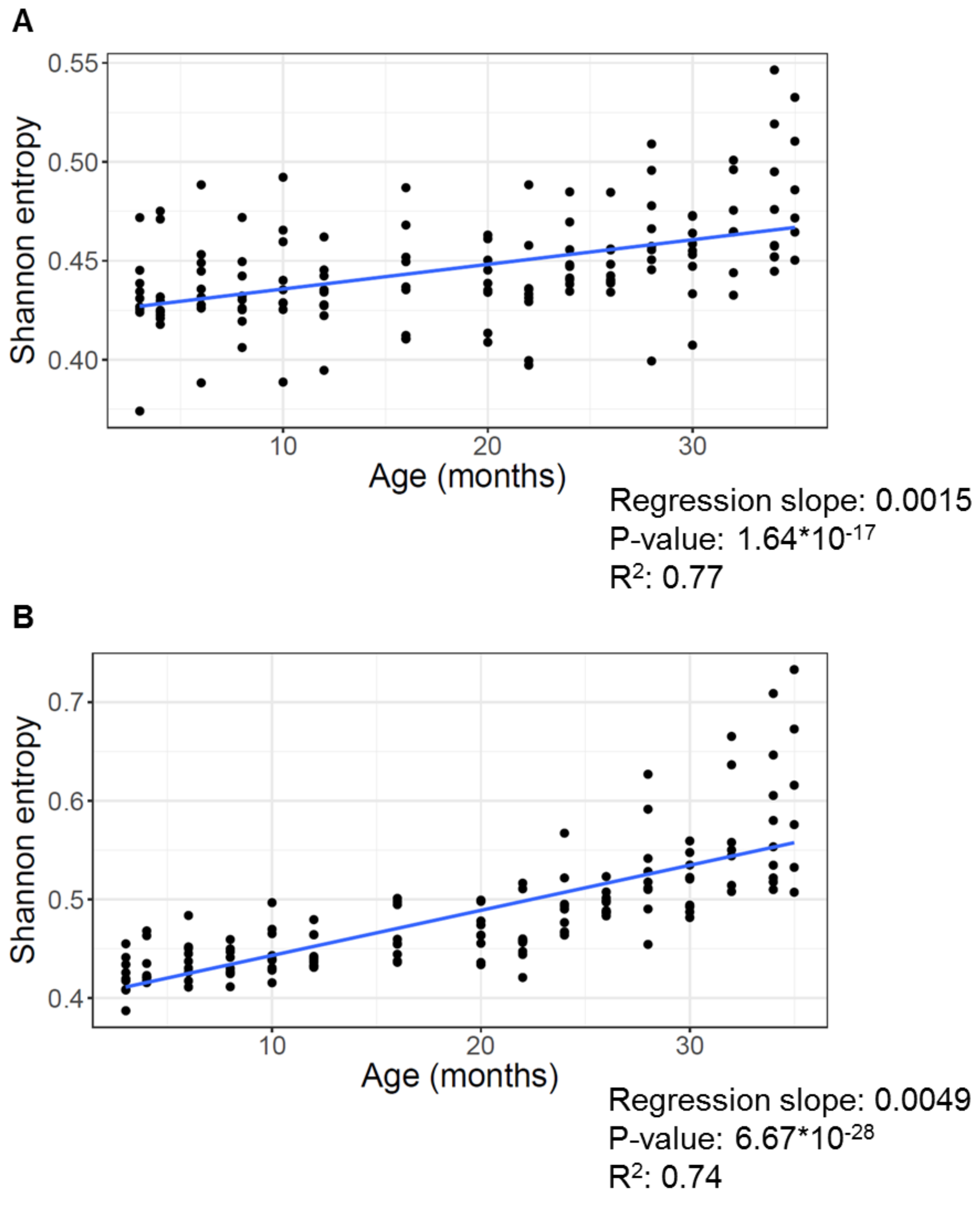

Supplementary Figure 14. Age-related increases in entropy of the DNA methylome. (A) Shannon entropy of 141 C57BL/6 mouse samples, calculated for every site. **(B)** Shannon entropy of the same samples, calculated only for the sites that significantly change with age.

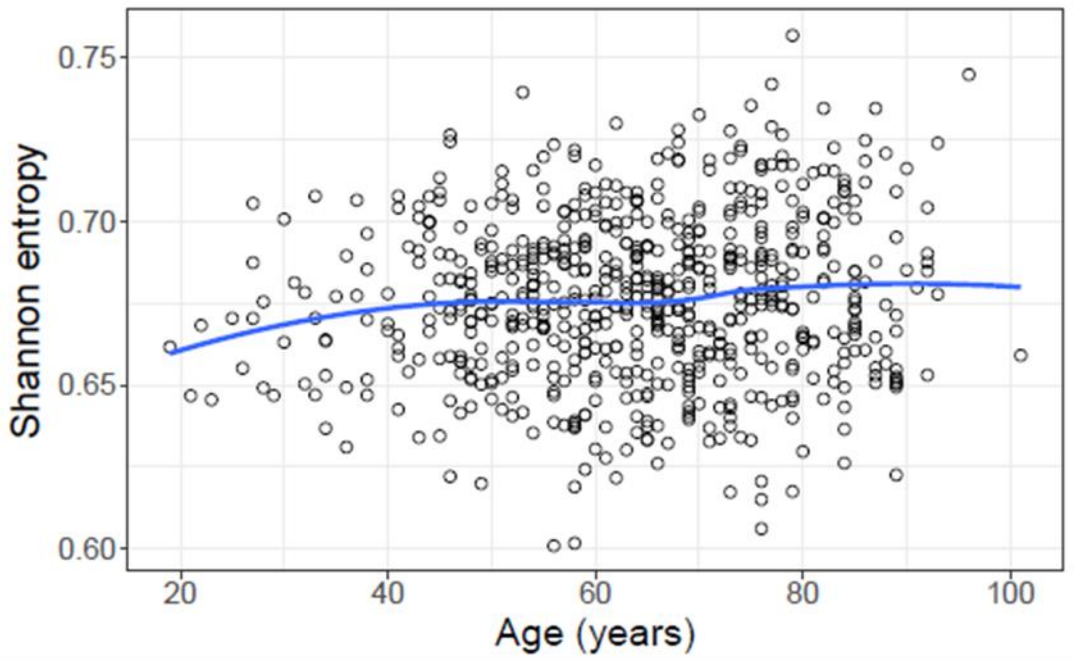

Regression slope: $3.98*10^{-5}$
P-value: 0.0063
Best fitting model: $age \sim entropy^{-\frac{1}{3}}$

Supplementary Figure 15. Age-related increases in entropy of the DNA methylome in human samples. Shannon entropy of 561 human samples, calculated for the sites that significantly change with age.

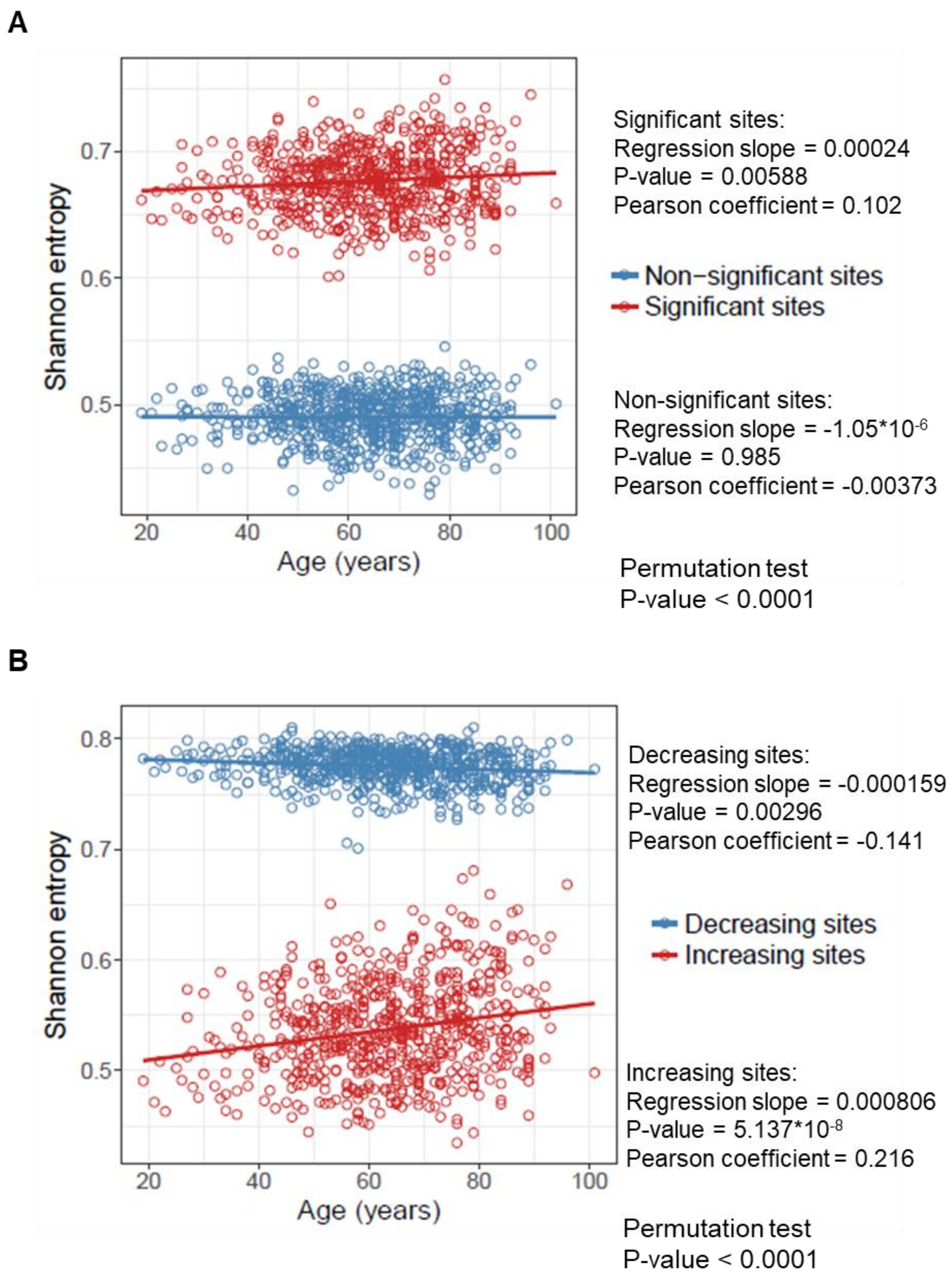

Supplementary Figure 16. Age-related changes in entropy of the human DNA methylome. (A) Shannon entropy of the sites that significantly change (or do not change) with age in 651 human samples from the age of 19 to 101 years. Permutation test was performed to assess the difference in entropy between changing and non-changing groups. **(B)** Shannon entropy of the sites that significantly increase and decrease with age in 651 human samples from the age of 19 to 101 years. Permutation test was performed to assess the difference in entropy for increasing and decreasing groups.

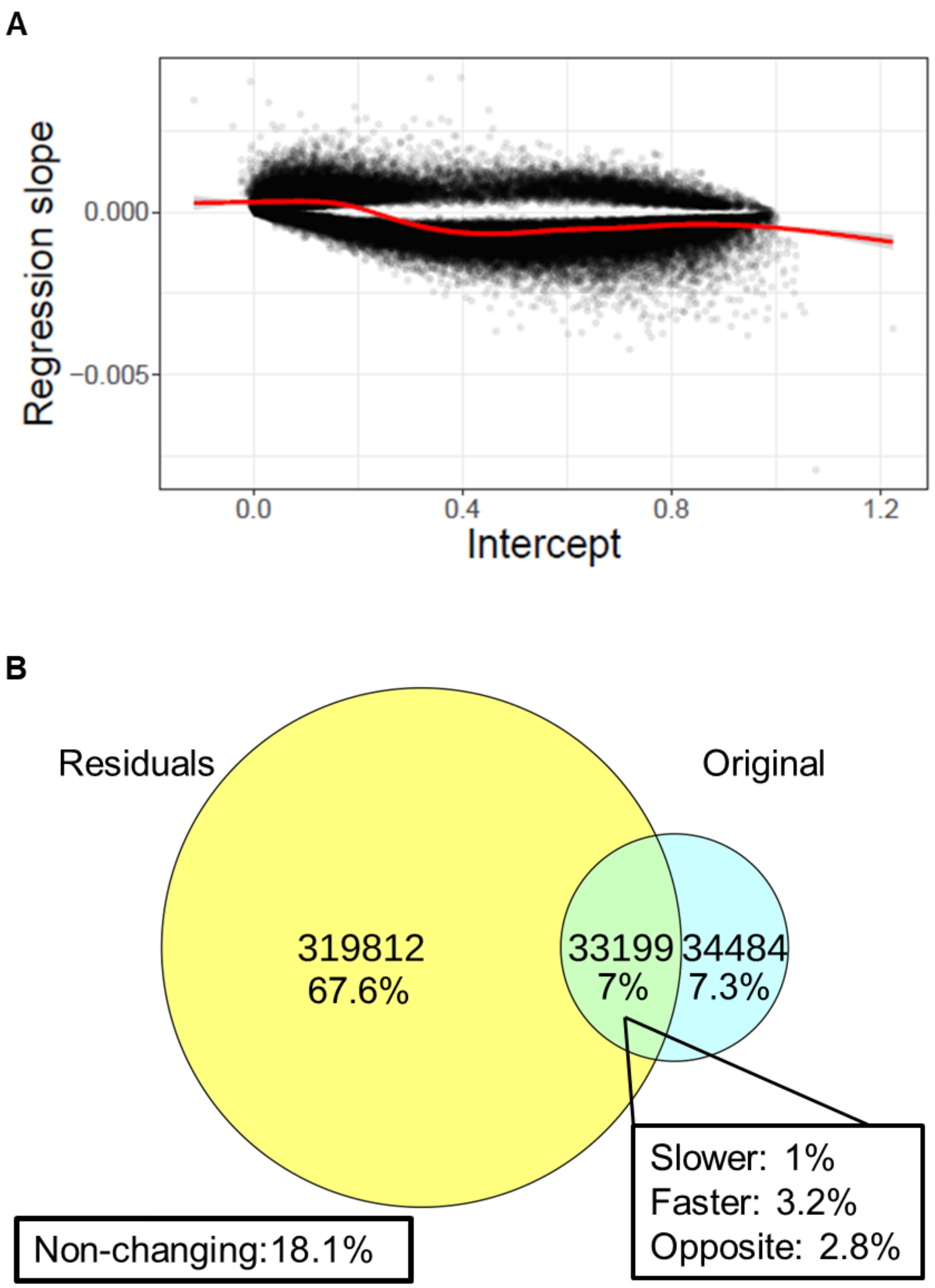

Supplementary Figure 17. Normalization for average entropy in 651 human samples. (A) Intercept of the linear regression versus the regression slope for every site that significantly changed with age in humans. The red curve represents generalized additive model fit. **(B)** Venn diagram of significantly changed sites, based on the original age-related changes (Original) and entropy-normalized residuals (Residuals) in human samples. Slower, faster and opposite represent the percentage of sites that change slower or faster than the average entropy or opposite to the one expected by entropy. Non-changing sites did not change in either of the analysis.

Supplementary Tables

Supplementary Table 1. Data transformation and multiple models.

Data transformations used	Number of significant sites	Percent of significant sites (%)	Sites gaining methylation (%)	Sites losing methylation (%)
$age \sim methylation^{-\frac{1}{3}}$	146067	85,9	41,6	44,3
$ln(age) \sim methylation^{-\frac{1}{3}}$	24059	14,1	6,5	7,6
$age \sim methylation$	0	0	0	0
$ln(age) \sim methylation$	0	0	0	0
$age \sim methylation^{2}$	0	0	0	0
$ln(age) \sim methylation^{2}$	0	0	0	0
$age \sim methylation^{3}$	0	0	0	0
$ln(age) \sim methylation^{3}$	0	0	0	0
$age \sim methylation^{\frac{1}{2}}$	0	0	0	0
$ln(age) \sim methylation^{\frac{1}{2}}$	0	0	0	0
$age \sim methylation^{\frac{1}{3}}$	0	0	0	0
$ln(age) \sim methylation^{\frac{1}{3}}$	0	0	0	0
$age \sim ln(methylation)$	0	0	0	0
$ln(age) \sim ln(methylation)$	0	0	0	0
$age \sim methylation^{2} + methylation$	0	0	0	0
$ln(age) \sim methylation^{2} + methylation$	0	0	0	0
$age \sim methylation^{-1}$	0	0	0	0
$ln(age) \sim methylation^{-1}$	0	0	0	0
$age \sim methylation^{-2}$	0	0	0	0
$ln(age) \sim methylation^{-2}$	0	0	0	0
$age \sim methylation^{-3}$	0	0	0	0
$ln(age) \sim methylation^{-3}$	0	0	0	0
$age \sim methylation^{-\frac{1}{2}}$	0	0	0	0
$ln(age) \sim methylation^{-\frac{1}{2}}$	0	0	0	0

Table shows the data transformations used and descriptive statistics for best fitting models based on Akaike information criterion for the significantly changing sites during aging.

Supplementary Table 2. Data transformation and multiple models in human samples.

Data transformations used	Number of significant sites	Percent of significant sites (%)	Sites gaining methylation (%)	Sites losing methylation (%)
$age \sim methylation^{-\frac{1}{3}}$	57218	81	32,3	48,7
$ln(age) \sim methylation^{-\frac{1}{3}}$	13389	19	9	9,9
$age \sim methylation$	0	0	0	0
$ln(age) \sim methylation$	0	0	0	0
$age \sim methylation^{2}$	0	0	0	0
$ln(age) \sim methylation^{2}$	0	0	0	0
$age \sim methylation^{3}$	0	0	0	0
$ln(age) \sim methylation^{3}$	0	0	0	0
$age \sim methylation^{\frac{1}{2}}$	0	0	0	0
$ln(age) \sim methylation^{\frac{1}{2}}$	0	0	0	0
$age \sim methylation^{\frac{1}{3}}$	0	0	0	0
$ln(age) \sim methylation^{\frac{1}{3}}$	0	0	0	0
$age \sim ln(methylation)$	0	0	0	0
$ln(age) \sim ln(methylation)$	0	0	0	0
$age \sim methylation^{2} + methylation$	0	0	0	0
$ln(age) \sim methylation^{2} + methylation$	0	0	0	0
$age \sim methylation^{-1}$	0	0	0	0
$ln(age) \sim methylation^{-1}$	0	0	0	0
$age \sim methylation^{-2}$	0	0	0	0
$ln(age) \sim methylation^{-2}$	0	0	0	0
$age \sim methylation^{-3}$	0	0	0	0
$ln(age) \sim methylation^{-3}$	0	0	0	0
$age \sim methylation^{-\frac{1}{2}}$	0	0	0	0
$ln(age) \sim methylation^{-\frac{1}{2}}$	0	0	0	0

Table shows the data transformations used and descriptive statistics for best fitting models based on Akaike information criterion for the significantly changing sites during aging in 651 human samples from the age of 19 to 101 years.
